# Essential gene deletions producing gigantic bacteria

**DOI:** 10.1371/journal.pgen.1008195

**Published:** 2019-06-10

**Authors:** Jeannie Bailey, Julie Cass, Joe Gasper, Ngoc-Diep Ngo, Paul Wiggins, Colin Manoil

**Affiliations:** 1 Department of Genome Sciences, University of Washington, Seattle, Washington, United States of America; 2 Department of Physics, University of Washington, Seattle, Washington, United States of America; The University of Texas Health Science Center at Houston, UNITED STATES

## Abstract

To characterize the consequences of eliminating essential functions needed for peptidoglycan synthesis, we generated deletion mutations of *Acinetobacter baylyi* by natural transformation and visualized the resulting microcolonies of dead cells. We found that loss of genes required for peptidoglycan precursor synthesis or polymerization led to the formation of polymorphic giant cells with diameters that could exceed ten times normal. Treatment with antibiotics targeting early or late steps of peptidoglycan synthesis also produced giant cells. The giant cells eventually lysed, although they were partially stabilized by osmotic protection. Genome-scale transposon mutant screening (Tn-seq) identified mutations that blocked or accelerated giant cell formation. Among the mutations that blocked the process were those inactivating a function predicted to cleave murein glycan chains (the MltD murein lytic transglycosylase), suggesting that giant cell formation requires MltD hydrolysis of existing peptidoglycan. Among the mutations that accelerated giant cell formation after ß-lactam treatment were those inactivating an enzyme that produces unusual 3->3 peptide cross-links in peptidoglycan (the LdtG L,D-transpeptidase). The mutations may weaken the sacculus and make it more vulnerable to further disruption. Although the study focused on *A*. *baylyi*, we found that a pathogenic relative (*A*. *baumannii*) also produced giant cells with genetic dependencies overlapping those of *A*. *baylyi*. Overall, the analysis defines a genetic pathway for giant cell formation conserved in *Acinetobacter* species in which independent initiating branches converge to create the unusual cells.

## Author summary

Although essential genes control the most basic functions of bacterial life, they are difficult to study genetically because mutants lacking the functions die. We have developed a simple procedure for creating bacteria in which different essential genes have been completely deleted, making it possible to analyze the roles of the missing functions based on the features of the dead cells that result. When genes needed for the production of the cell wall were inactivated, the bacteria formed bizarre giant cells. It was possible to identify the functions responsible for forming the giant cells, and to formulate a model for how they form. Since cell wall synthesis is one of the most important antibiotic targets, understanding how bacteria respond to its disruption may ultimately help in developing procedures to overcome antibiotic resistant bacterial infections.

## Introduction

In spite of controlling the most fundamental biological processes, essential genes are usually missing from loss-of-function mutant screens because strains carrying null mutations are not represented. Although essential functions can be studied using conditional alleles, such as temperature-sensitive or regulated expression alleles, suitable alleles that support normal growth under permissive conditions while fully eliminating activity under non-permissive conditions can be difficult to isolate [1, 2]. In the work reported here, we describe the use of gene deletions generated by natural transformation as an alternative to conditional alleles for studying essential functions. We employed the approach to examine the consequences of inactivating genes needed for peptidoglycan synthesis in *Acinetobacter baylyi*, a Gram-negative bacterium belonging to the Gammaproteobacteria [3]. We focused on peptidoglycan because it is the major determinant of cell shape and mutations affecting it can produce dramatically altered cell morphologies [4–6].

**Fig 1:**
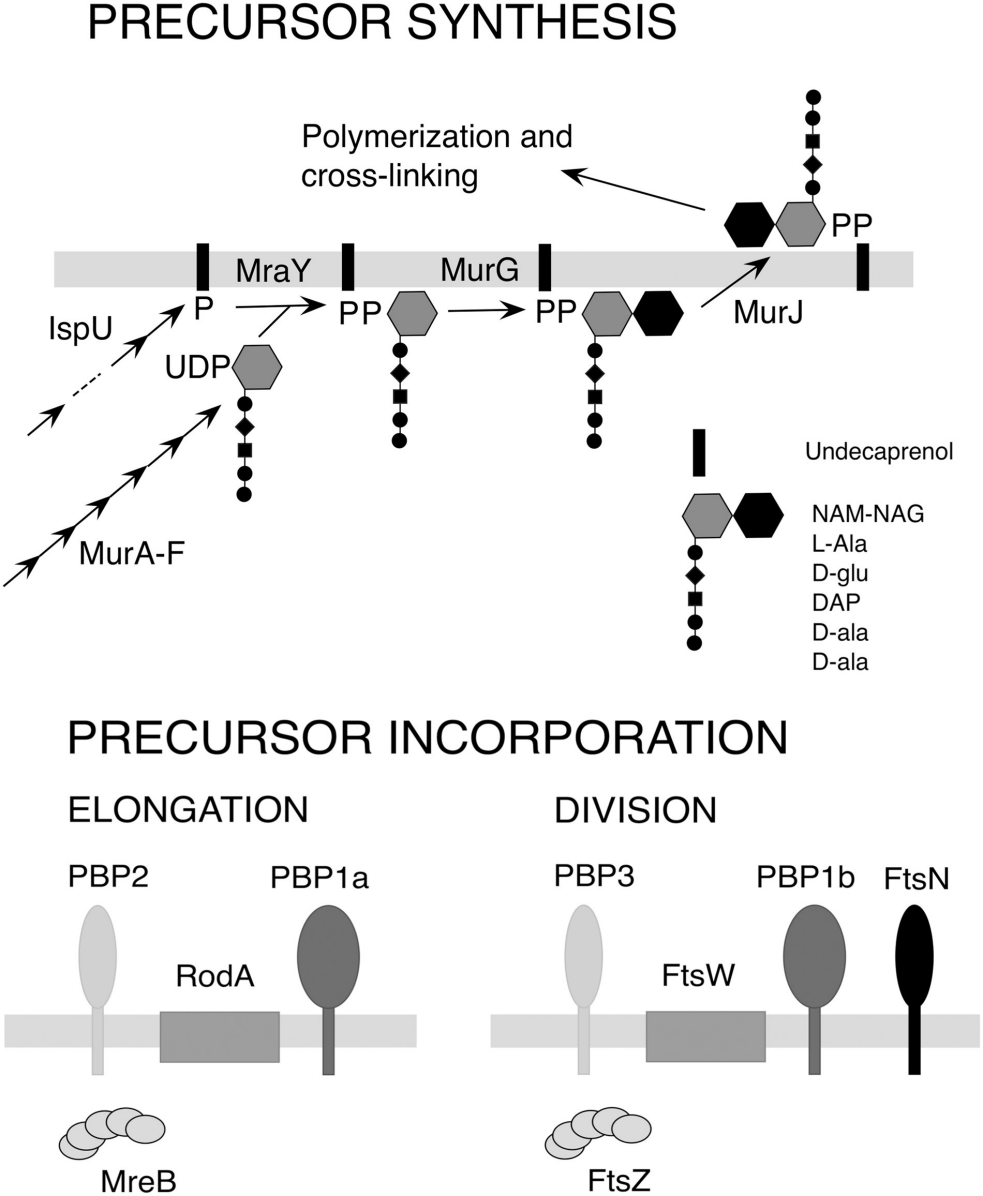
Peptidoglycan synthesis. Top panel, pathway of precursor undecaprenol disaccharide pentapeptide synthesis. Bottom panel, key functions in peptidoglycan elongation and septal synthesis [11]. NAM-NAG, N-acetyl muramyl-N-acetyl glucosamine; PBP2 (PbpA) and PBP3 (FtsI), transpeptidases; RodA and FtsW, transglycosylases; PBP1a (PonA) and PBP1b (MrcB), transglycosylase-transpeptidases; FtsN, division regulator; MreB and FtsZ, cytoskeletal proteins.

Peptidoglycan is an essential glycopeptide mesh situated between the two membranes of Gram-negative bacteria, which helps protect cells from lysis due to turgor pressure. Peptidoglycan is constructed by complex mechanisms in which a lipid-linked disaccharide pentapeptide precursor is incorporated into peptidoglycan through the action of transglycosylase and transpeptidase activities (Fig 1). Parallel sets of enzymes are required for cell elongation and division [7–11].

Peptidoglycan growth reflects a balance between synthetic and degradative activities [7, 8]. When new peptidoglycan biosynthesis is blocked, hydrolytic activities degrade the existing sacculus and bacteria usually lyse due to osmotic swelling. However, under osmoprotective conditions, cells are prevented from lysing and may proliferate as pleiomorphic wall-deficient cells called L-forms [12–15]. These bacteria have been an object of fascination for decades due to their capacity for growth without a peptidoglycan sheath, striking cell morphologies and antibiotic resistance [14, 15]. It appears that the formation of L-forms requires not only loss of peptidoglycan synthesis, but also additional mutations that allow the wall-deficient forms to proliferate by membrane tubulation and blebbing [5].

In some cases, loss of peptidoglycan synthesis leads not to L-forms, but to non-proliferating cells that have lost their normal shape and may enlarge considerably [5, 12, 13, 16–19]. For example, in one well-characterized example, mutations inactivating an *E*. *coli* cytoskeletal protein needed for elongation peptidoglycan synthesis (MreB) indirectly inactivate division by sequestering a division protein (FtsZ) in internal membranes [13], leading to giant cells. In other cases, blocking peptidoglycan synthesis converts the bacteria into small non-dividing spherical cells [16, 17, 20]. In *Vibrio cholera*, formation of such spheroids requires murein endopeptidase activity [16]. The small spherical cells can grow when peptidoglycan synthesis resumes; their formation thus represents a novel antibiotic tolerance mechanism. Genetic determinants of this tolerance have been defined [20].

In this study, we examined the consequences of disrupting peptidoglycan synthesis in *Acinetobacter baylyi*. There are several advantages of studying peptidoglycan synthesis in this bacterium. First, the species undergoes natural transformation at high efficiency [21, 22], making it straightforward to generate deletion mutations in essential genes. Second, peptidoglycan synthesis associated with cell elongation is fully dispensable, making it simple to genetically manipulate septal synthesis in the absence of elongation synthesis. Third, like *E*. *coli*, *A*. *baylyi* belongs to the Gammaproteobacteria, and the detailed understanding of peptidoglycan synthesis and cell division in *E*. *coli* should provide a good foundation for understanding the processes in *A*. *baylyi*. Finally, *A*. *baylyi* peptidoglycan metabolism should be similar to that of the related nosocomial pathogen *Acinetobacter baumannii*. Understanding the consequences of disrupting peptidoglycan synthesis in *A*. *baylyi* may thus suggest approaches for enhancing the efficacy of antibiotics targeting the process in its clinically relevant relative.

We found that *A*. *baylyi* forms giant cells in response to a variety of deletion mutations and antibiotics blocking peptidoglycan synthesis. We exploited the powerful genetic analysis possible in *A*. *baylyi* to characterize the requirements for formation of giant cells and to formulate a pathway for their creation.

## Results and discussion

### Essential gene deletion mutations

**Fig 2:**
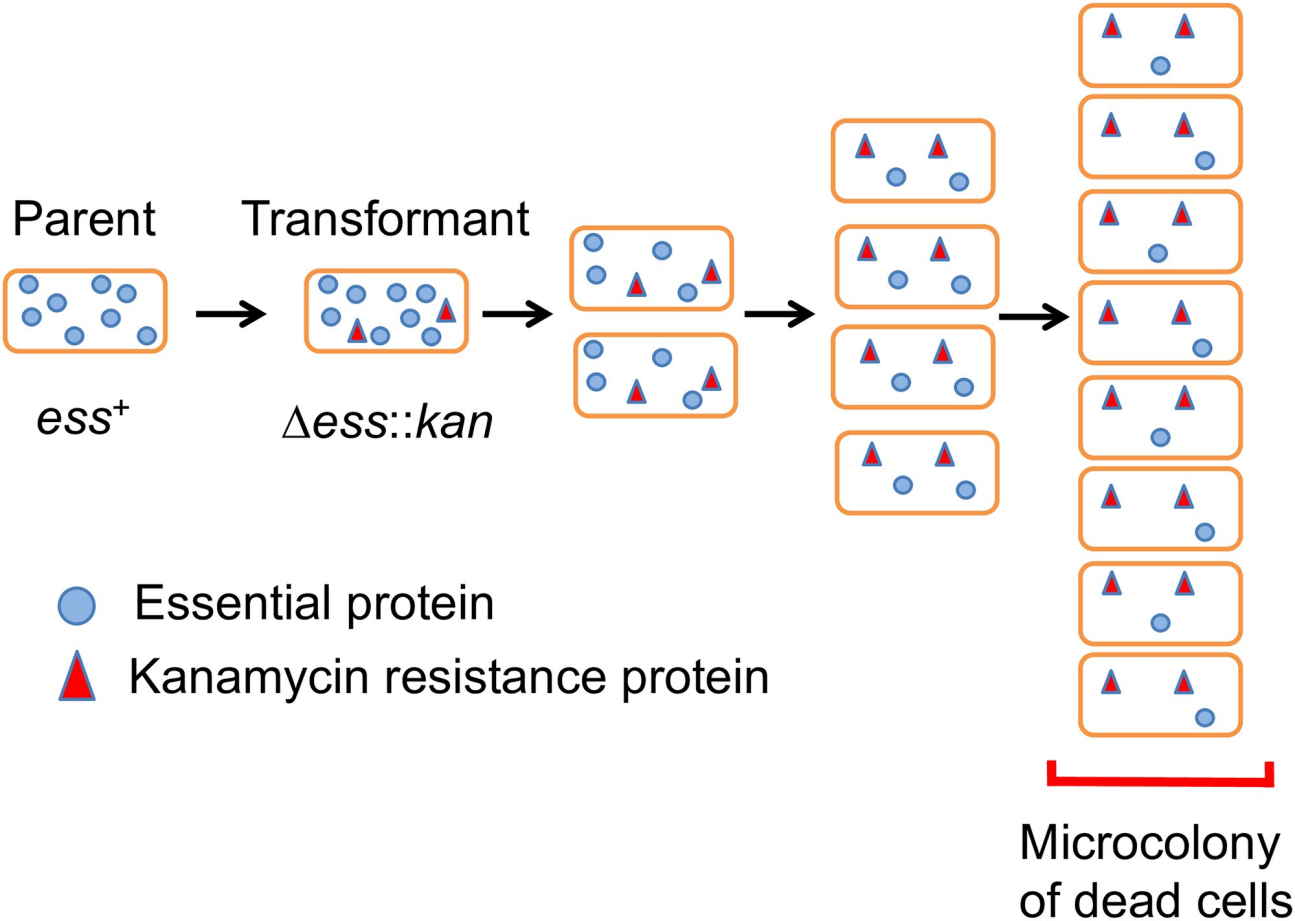
Cellular depletion of an essential product following gene deletion. Replacement of an essential gene with a kanamycin resistance marker allows growth and division of cells on agar medium supplemented with kanamycin until the essential product is sufficiently depleted to block further growth. The properties of the microcolony of dead cells that results reflect the phenotypic consequences of the deletion mutation. *ess*, essential gene; *kan*, kanamycin resistance determinant.

We sought to characterize the cellular consequences of disrupting conserved essential processes like peptidoglycan synthesis. To do this, we generated essential gene deletions by natural transformation of *A*. *baylyi* and examined the cells that resulted. Bacteria were exposed to PCR DNA fragments that replace targeted genes with a kanamycin resistance determinant, followed by plating on agar containing kanamycin (Fig 2) (Materials and methods). Transformed cells incorporate the mutagenic DNA, deleting the corresponding essential gene, and then grow and divide, depleting the essential product. Proliferation stops when cells run out of the targeted essential gene product. The resulting microcolonies are made up of dead cells whose morphologies reflect loss of the targeted essential product, and whose size reflects how rapidly depletion of the product blocks growth. Typically 5–10% of the cells are transformed to generate deletions in such experiments, and kanamycin-sensitive untransformed cells are readily distinguished because they stop dividing largely as singlets and doublets with vegetative cell morphology (see below). The principle unwanted background event in the generation of essential gene deletions (occurring at ~10^−6^ frequency) appeared to be due to transformation of partially diploid cells (presumably due to tandem duplications), which generated fast growing cells carrying copies of both deletion and wild type alleles of targeted genes (Materials and methods) [23–26].

### Mutations blocking peptidoglycan precursor synthesis lead to the formation of giant cells

**Fig 3:**
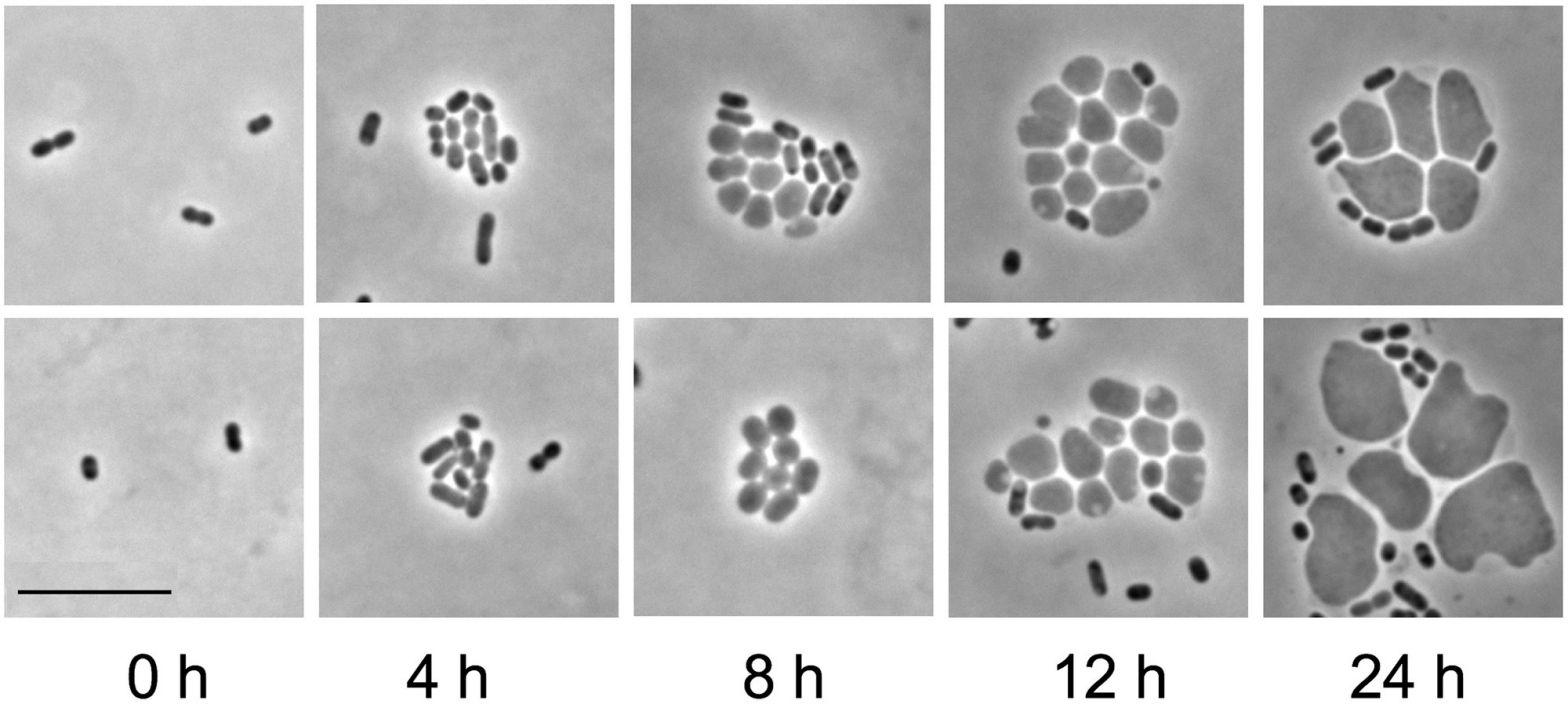
Microcolonies of giant cells formed after *murA* deletion. Two representative fields of developing microcolonies at different times following deletion of *murA* from wild type (MAY101) are shown. Cells were transformed with a Δ*murA*::*kan* PCR fragment, followed by incubation on agarose pads containing protective medium and kanamycin. Giant cell microcolonies usually contained some cells with vegetative cell dimensions. Such cells may originate from transformants that carried multiple copies of the *murA* locus (e.g., in multiple or partially replicated chromosomes), not all of which incorporated a Δ*murA*::*kan* fragment. Scale bar, 10 μm.

In a large-scale screen of microcolonies of cells carrying deletion mutations affecting different essential processes, the most dramatic phenotypes resulted from disruption of peptidoglycan synthesis (Fig 1). For example, when the gene encoding the first step of lipid-linked disaccharide pentapeptide precursor synthesis (*murA*) was deleted by transformation with a Δ*murA*::*kan* PCR fragment, microcolonies of polymorphous giant cells formed (Fig 3). The giant cells were stabilized by high osmolarity medium and typically enlarged for ~12–24 hours. Giant cells also formed when wild-type bacteria were treated with fosfomycin, an antibiotic that targets MurA (see below). Giant cells could reach diameters greater than ten times that of vegetative cells (see below), and often contained one or more vacuoles at their peripheries (Fig 3, 12 and 24 h). The vacuoles failed to fluoresce in cells expressing cytoplasmic green fluorescent protein (S1 Fig), indicating that they are shielded from the cytoplasm, e.g., as if they were derived from the periplasm. Developing giant cells were sometimes joined to each other by membranous bridges (Fig 3, 8 h bottom panel) or exhibited wispy filaments with vesicles extending from their surfaces (S1 Fig). The giant cells did not proliferate, and thus are distinct from L forms.

Time-lapse imaging of giant cell formation following *murA* deletion shows a process in which cells transform from rods into amorphous amoeboid cells that enlarge and eventually burst (S1 Movie). The development of the giant cells proceeds by enlargement without apparent midcell (preseptal) blebbing [27].

**Fig 4:**
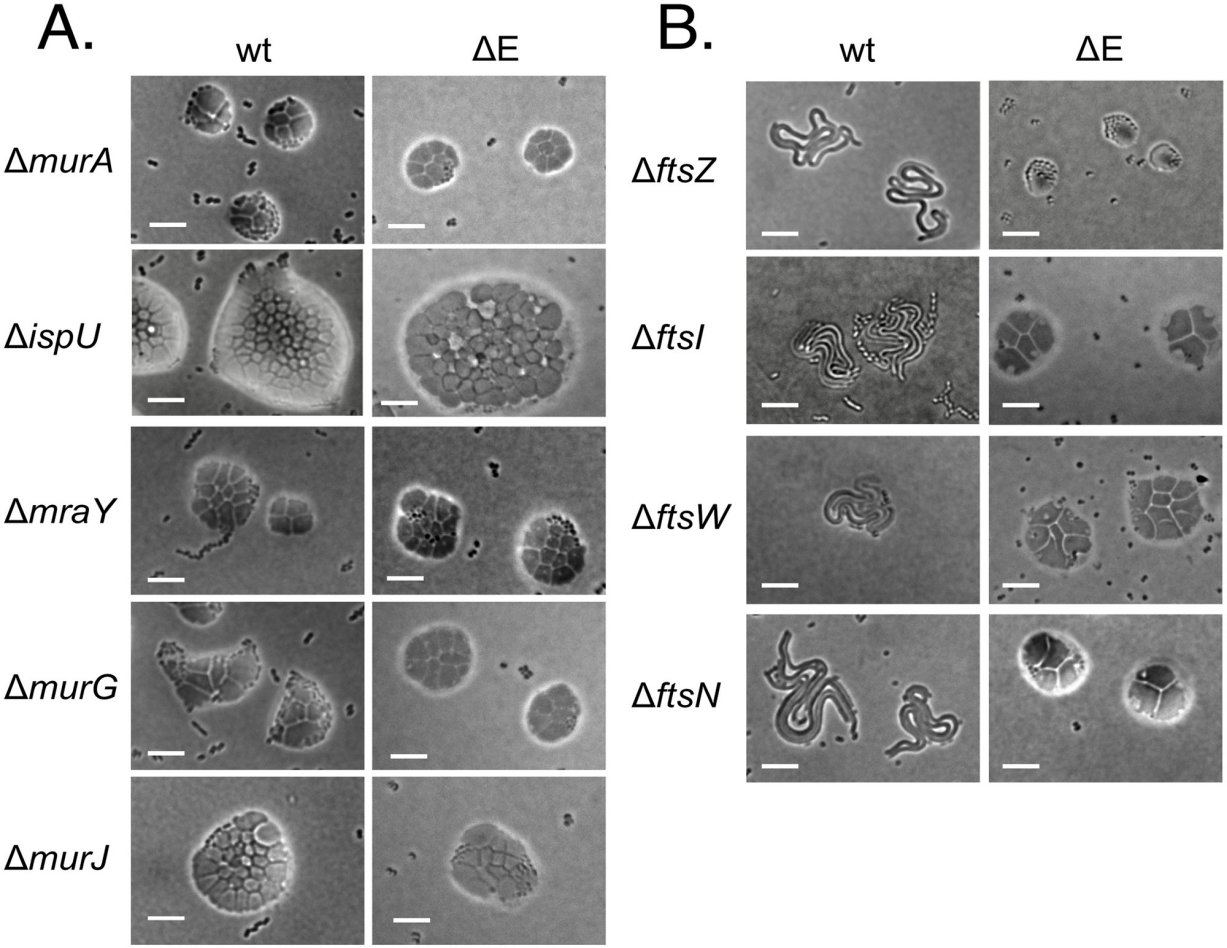
Blocking peptidoglycan precursor synthesis or polymerization leads to giant cells. **A, Precursor synthesis mutants.** Microcolonies formed after deletion of genes required for different steps of peptidoglycan precursor synthesis are shown. Deletions of *meso*-diaminopimelic acid synthesis genes *dapA* and *dapB* also resulted in giant cells. Colonies were incubated 20–24 h on protective agar. Scale bar, 10 μm. **B, Microcolonies of cell division mutants.** Microcolonies formed after deletion of genes required for cell division are shown. Colonies were incubated 20–24 h on protective agar. wt, wild-type (MAY101) genetic background; ΔE, peptidoglycan elongation-minus triple mutant (*ΔpbpA ΔrodA ΔponA*) genetic background (MAY106). Scale bar, 10 μm.

Deletions of other genes required for peptidoglycan precursor biosynthesis also produced microcolonies of giant cells (Fig 4A). The microcolonies appeared similar in wild type (wt) and in a peptidoglycan elongation–minus triple mutant genetic background (“ΔE”) (see below). Mutations in three of the genes (*murG*, *murJ* and *mraY)* led to microcolonies similar in size to those produced by Δ*murA* mutants. The deletion of the fifth gene (*ispU*), required for cofactor undecaprenol synthesis, led to larger microcolonies than the others. Since undecaprenol is recycled rather than consumed by peptidoglycan synthesis, it may require more growth to deplete it than the precursor intermediates, leading to the larger mutant microcolonies. Overall, the results indicate that disrupting peptidoglycan precursor synthesis at different steps has a similar consequence, the formation of small microcolonies of giant cells. This phenotypic consistency suggests that intermediates in the peptidoglycan precursor synthetic pathway are not particularly toxic, in contrast, for example, to the lipopolysaccharide biosynthetic pathway [28].

### Mutations blocking peptidoglycan precursor polymerization also produce giant cells

The lipid-linked disaccharide pentapeptide precursor is incorporated into peptidoglycan through the action of transglycosylase and transpeptidase activities, with different machineries responsible for cell elongation and division (Fig 1) [7]. Enzymes making up the elongation complex are nonessential in *A*. *baylyi*, although elongation–minus mutants grow as spheres rather than short rods [25]. Septal peptidoglycan synthesis functions are essential in *A*. *baylyi*. To evaluate whether blocking disaccharide pentapeptide precursor incorporation into peptidoglycan led to giant cell formation, we created mutants defective in both elongation and septal peptidoglycan synthesis. These strains were created from a parent (“ΔE”; MAY106) carrying deletion mutations eliminating three elongation functions (PBP2, RodA and PBP1a), combined with different mutations blocking cell division (Fig 4B). In all cases, elimination of elongation and division functions together led to the formation of giant cells, whereas the division mutations alone led to long filaments (Fig 4B). The results show that like eliminating precursor synthesis, disrupting incorporation of the precursors into peptidoglycan produces giant cells.

### Antibiotics targeting peptidoglycan synthesis also induce giant cell formation

**Fig 5:**
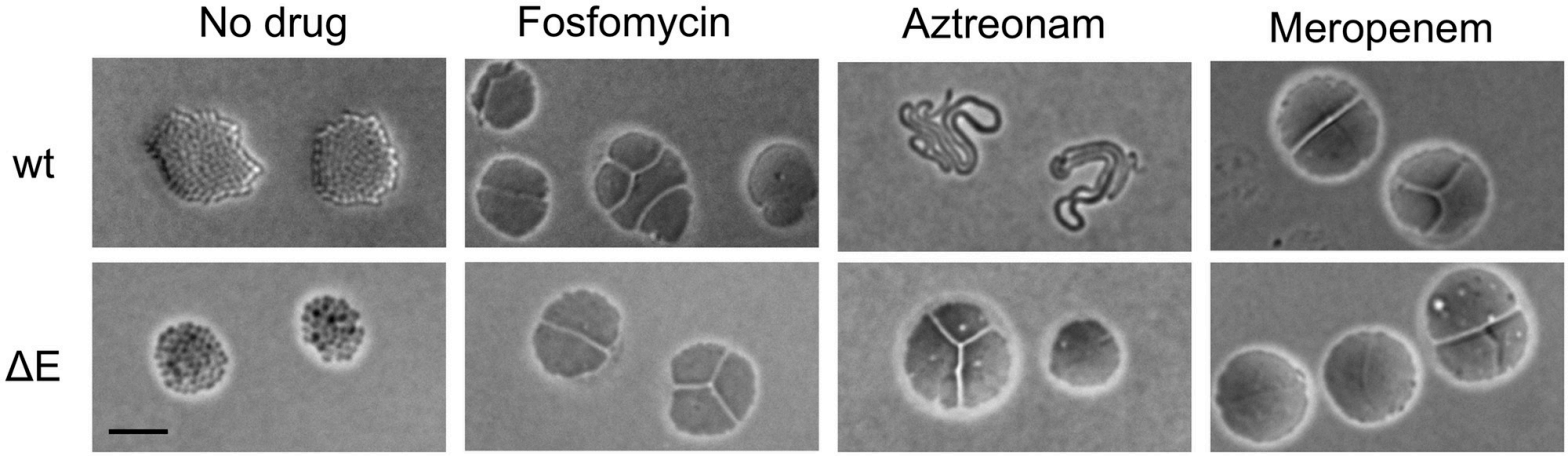
Antibiotic treatments producing giant cells. Microcolonies were grown on protective agar containing the indicated antibiotics (24 h) or no drug (11 h). The antibiotic concentrations are: fosfomycin (192 μg/ml), aztreonam (192 μg/ml) and meropenem (5 μg/ml). WT, wild type (MAY101); ΔE, peptidoglycan elongation-minus triple mutant (MAY106). Scale bar, 10 μm.

We also examined whether treating cells with antibiotics targeting peptidoglycan synthesis produced giant cells. In agreement with the mutant studies, we found that antibiotics inhibiting precursor synthesis or peptide cross-linking induced giant cells (Fig 5). The antibiotics examined were fosfomycin, which targets MurA, aztreonam, which targets the division-specific transpeptidase FtsI (PBP3), and meropenem, which apparently targets both the PbpA (PBP2) and FtsI (PBP3) transpeptidases [29]. As expected, fosfomycin and meropenem treatments induced giant cells in both wild type and in an elongation-minus genetic background, whereas aztreonam induced giant cells only in the elongation-minus background. For all three antibiotic treatments, the giant cells were comparable in size, with median dimensions ~10 times that of untreated cells (Table 1).

**Table 1:** Giant cell size. Dimensions of untreated cells or cells grown for 20–24 hr, 30° C, on protective agar containing fosfomycin (192 μg/ml), aztreonam (192 μg/ml) (*pbpA* deletion mutant), or meropenem (10 μg/ml) were measured. Isolated cells or cells in small microcolonies (≤4 cells) were included. W, width; L, length; D, diameter.

Treatment	Median size (μm)	Range (μm)	Number
None (WT)	0.6 (W) X 1.2 (L)	0.4–0.9 (W) X 0.8–2.1 (L)	100
None (Δ*pbpA*)	1.2 (D)	0.7–1.6 (D)	100
Fosfomycin (WT)	9.3 (D)	1.8–13.7 (D)	82
Aztreonam (Δ*pbpA*)	12.1 (D)	2.9–15.3 (D)	55
Meropenem (WT)	8.2 (D)	2.9–12.6 (D)	150

Treatments with two additional antibiotics, cycloserine, which targets Ddl (D-ala-D-ala racemase) and Alr (alanine racemase), and the ß-lactam mecillinam, which apparently targets multiple cross-linking enzymes in *A*. *baylyi* (unlike in *E*. *coli*) [11], also led to giant cells. The findings indicate that, as was seen for deletion mutations, antibiotic inhibition of peptidoglycan precursor synthesis or incorporation into the sacculus produces giant cells.

### Mutations altering giant cell formation

A straightforward model for giant cell formation is that after inhibition of peptidoglycan synthesis blocks cell enlargement, the activity of hydrolytic functions ruptures the peptidoglycan sheath, allowing the growing cytoplasm to break out of it and expand [5, 13]. To identify functions potentially involved in this process, we screened for mutations altering giant cell recovery using saturation-level transposon mutant sequencing (Tn-seq). We assumed that the representation of mutations that either blocked or accelerated giant cell formation and subsequent lysis would be changed compared to growth without giant cell induction.

We carried out the screens after inducing giant cell formation by fosfomycin or aztreonam treatment (Materials and methods). For the fosfomycin treatment screens, we created a genome saturation-level mutant pool in wild type by transposon-transposase complex electroporation mutagenesis [30]. The pool was exposed to fosfomycin on protective medium to induce giant cells, and DNA isolated from the cells after 24 h growth was subjected to Tn-seq (Materials and methods). Mutations in 35 genes reduced recovery and in 56 genes increased recovery in the presence of fosfomycin compared to no antibiotic (S1 Database). A second set of Tn-seq screens employed a *ΔpbpA* (PBP2) mutant pool created by natural transformation of the wild-type pool used for the fosfomycin screen by *ΔpbpA*::kan (Materials and methods). The *ΔpbpA* mutant pool was exposed to aztreonam on protective medium, and DNA isolated at two different times for Tn-seq. In these screens, mutations in 54 genes reduced recovery and in 27 genes increased recovery relative to no treatment (S1 Database). Among the mutations depleted in one or both screens were those inactivating peptidoglycan penicillin binding proteins, recycling functions, and other proteins involved in peptidoglycan metabolism.

Two caveats in interpreting the phenotypes of transposon insertion mutants are that strains may carry unlinked mutations, and that insertions may have polar effects on downstream genes in operons. Unlinked mutations responsible for phenotypes are unlikely in our analysis because the phenotypes identified in the Tn-seq screens are seen for multiple independent insertions (>35/gene on average). In most cases, polar effects are also unlikely to account for mutant phenotypes, because the saturation-level genome coverage Tn-seq provides includes all of the (nonessential) genes in an operon. Thus, a polar effect would be seen as a downstream gene (as well as the upstream gene) having a mutant phenotype. Although there were four such genes in our top set, validation studies for three of them with constructed mutants designed to be nonpolar indicate that their phenotypes were not due to polarity (next section).

### Validation of Tn-seq findings

Since Tn-seq assays involve cells grown in competition, weak growth phenotypes can lead to significant mutant representation changes. To distinguish the subset of genes with strong mutant phenotypes, we carried out validation experiments with individual mutants. We constructed and analyzed 38 deletion mutants corresponding to genes identified in the Tn-seq screens (32/38) or considered candidates based on their annotated functions (6/38). The mutations were created by replacing the targeted genes with a kanamycin resistance determinant oriented the same as the deleted gene to support transcription of any downstream genes and reduce polar effects [1] (Materials and methods). A total of 29 of the 38 deletions were confirmed to affect giant cell formation (S1 Table), eleven of them leading to particularly strong phenotypes (Table 2).

**Table 2:** Mutations altering giant cell formation. The genes with the strongest mutant phenotypes among the deletion mutants examined (S1 Table) are listed. The number/total Tn-seq runs in which mutants in the indicated genes were significantly depleted or enriched are indicated. PBP, penicillin binding protein; nd, not determined due to low representation in the ΔPBP2 transposon mutant pooled library; wt, wild type.

Locus	Gene	Product	Fosfomycin (wt)		Aztreonam (ΔPBP2)	
			Tn-seq recovery	Deletion phenotype	Tn-seq recovery	Deletion phenotype
ACIAD0527	*gcf*	Sel1 domain	Decreased (2/2)	Fails to form giant cells, early lysis	nd	Early lysing clusters of small giant cells
ACIAD0551	*nagZ*	N-acetyl-ß-glucosaminidase	Decreased (2/2)	Early forming, early lysing giant cells	Decreased (7/8)	Slightly early lysing giant cells
ACIAD1138	*mltD*	Lytic transglycosylase	Decreased (2/2)	Fails to form giant cells, early lysis	–	Early lysing small giant cells
ACIAD1225	*dacA*	PBP5	–	Forms giant cells	Decreased (8/8)	Early forming, early lysing giant cells
ACIAD1396	*–*	Histidine triad protein	Increased (2/2)	Late forming, late lysing giant cells	Increased (8/8)	Partial resistance
ACIAD2234	*mrcB*	PBP1b	Decreased (2/2)	Fails to form giant cells, early lysis	Decreased (2/8)	Early forming, early lysing giant cells
ACIAD2235	*lpoB*	PBP1b activator	Decreased (2/2)	Fails to form giant cells, early lysis	Decreased (1/8)	Forms giant cells
ACIAD2336	*zapE*	Divisome ATPase	Increased (2/2)	Late forming, late lysing giant cells	Increased (8/8)	Partial resistance
ACIAD2475	*ldtG*	L, D-transpeptidase	–	Forms giant cells	Decreased (8/8)	Early forming, early lysing giant cells
ACIAD3361	*ponA*	PBP1a	–	Forms giant cells	Decreased (7/8)	Early forming, early lysing giant cells
ACIAD3380	*ettA*	Translation regulator	Increased (2/2)	Late forming stable giant cells	Increased (5/8)	Partial resistance

**Fig 6:**
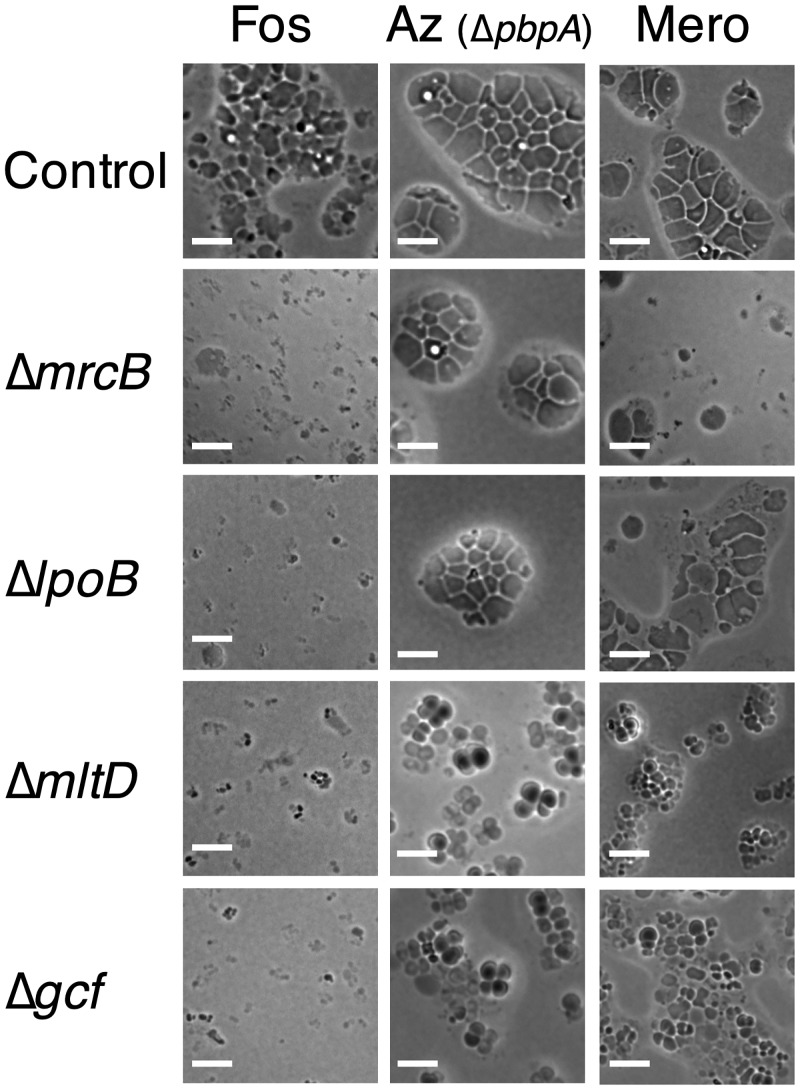
Mutations blocking giant cell formation in *A*. *baylyi*. The microcolonies formed by mutants blocked in giant cell formation on protective agar containing antibiotics inducing giant cell formation are shown. Mutations were in the wild type genetic background (MAY101) for the fosfomycin and meropenem treatments and in the PBP2^−^ (Δ*pbpA*) genetic background (MAY102) for the aztreonam assays. Control, ΔIS (MAY116); Fos, fosfomycin (360 μg/ml); Az, aztreonam (192 μg/ml); Mero, meropenem (10 μg/ml). Scale bar, 10 μm.

Mutations in four of the eleven genes blocked giant cell formation at intermediate stages. Deletions of two of the four (*mrcB*, encoding transglycosylase-transpeptidase PBP1b, and *lpoB*, encoding an MrcB regulator) [31], had similar phenotypes, leading to much stronger defects in fosfomycin induction than aztreonam induction, with intermediate effects on meropenem induction (Fig 6). The mutations blocked fosfomycin induction at an early stage at which cells rounded but did not enlarge significantly before lysing. Deletions of the other two genes (*mltD*, encoding a membrane lytic transglycosylase, and *gcf (*giant cell formation), encoding an exported protein of unknown function) blocked induction by all three antibiotics. However, the phenotypes differed depending on the condition. The blocks in fosfomycin induction were severe, with modest but detectable enlargement before lysis. In contrast, both mutations allowed more enlargement following aztreonam and meropenem induction, producing clusters of “small giants”. Bridges between cells in such clusters were common. Similar clusters were seen as an intermediate in the formation of giant cells after aztreonam treatment of the ΔPBP2 strain.

Mutations in four other genes accelerated giant cell formation and lysis. Mutations in three of them were specific to aztreonam induction (*ldtG*, encoding a peptidoglycan DAP-DAP (3->3) cross-linking enzyme; *dacA*, encoding peptidoglycan carboxypeptidase PBP5; and *ponA*, encoding transglycosylase-transpeptidase PBP1a) (S2 Fig). We suspected that the mutations may lead to a weakened peptidoglycan with reduced cross-linking that is more sensitive to further reduction upon aztreonam exposure. Indeed, all three mutations reduced the aztreonam minimal inhibitory growth concentration (MIC) (S2 Fig legend). Mutations in a fourth gene (*nagZ*, encoding a peptidoglycan recycling function) led to a complex phenotype. They affected fosfomycin induction more strongly than aztreonam induction, with some early giant cell formation and rampant lysis (S3 Fig). Other recycling pathway mutants also reduced giant cell recovery in Tn-seq (S1 Database). The recycling pathway provides a precursor that bypasses the fosfomycin-inhibited step of the *de novo* pathway (MurA). As thus expected, the *nagZ* mutation reduced the fosfomycin MIC (S3 Fig legend).

Most mutations increasing Tn-seq recovery moderately to severely reduced vegetative growth rate and may simply delay giant cell lysis by slowing down progression through the pathway (S1 Database). Three examples of such genes with strong mutant phenotypes were *ettA*, encoding a regulator of translation, *ACIAD1396*, encoding a histidine triad protein of unknown function, and *zapE*, encoding a division ATPase (Table 2). We did not identify any mutations allowing giant cells to propagate as L forms.

In the course of these validation experiments, we identified an unusual phenotype associated with loss of *zipA*, a gene encoding a division protein that is essential in *E*. *coli* but not in *Acinetobacter* species [32]. A Δ*zipA* mutation alone caused *A*. *baylyi* cells to propagate as elongated rods, some very long (S4 Fig). When a Δ*zipA* mutation was combined with a mutation blocking elongation peptidoglycan synthesis (ΔPBP2), the double mutant bacteria propagated as mixed colonies of enlarged spherical cells and giant cells (S4 Fig). We suspect that the *zipA* mutation hobbles septal peptidoglycan synthesis such that Δ*zipA* ΔPBP2 double mutant cells divide less frequently than ΔPBP2 mutant alone, leading to larger spheres, and that occasional outright division failure in the double mutant leads to the production of the giant cells.

### Lipooligosaccharide-minus mutants form fragile giant cells

*Acinetobacter* species produce an outer membrane lipooligosaccharide (LOS) that corresponds to lipopolysaccharide lacking an O side chain [33]. Since the outer membrane contributes significantly to envelope stability [34], we sought to examine whether lipooligosaccharide (LOS) is needed for giant cell formation. Although LOS is nonessential in *Acinetobacter* species [33], its absence slows growth and the corresponding mutants were poorly represented in the transposon mutant pools we used for Tn-seq analysis. To test the requirement for LOS in *A*. *baylyi* giant cell formation, we therefore generated deletions of genes required for LOS precursor synthesis (*lpxA)* or transport to the outer membrane (*lptAB*). Both classes of mutations affected giant cell formation. The LpxA mutant, which grew as enlarged spheres without antibiotic, formed giant cells after fosfomycin treatment that frequently lysed earlier than usual (S5 Fig). The LptAB deletion mutant grew as smaller spheres and had a more dramatic defect, with massive lysis under giant cell induction conditions. Recent studies have found that LOS transporter mutations cause toxic precursors to accumulate in cells [28, 35], and we suspect the Δ*lptAB* phenotype may be accentuated by such toxicity. Overall, the studies thus indicate that the loss of LOS does not block giant cell formation, but that the cells formed are more fragile than those of wild type.

### Reverse phenocopy test

If the antibiotics that induce giant cell formation act by inhibiting their established targets (MurA for fosfomycin and FtsI (PBP3) for aztreonam), inducing giant cells by deleting the target genes should show the same genetic dependencies as those seen for antibiotic induction. To test this prediction, we examined whether three of the mutations blocking giant cell formation after antibiotic treatment also blocked it after deletion of the target genes. We saw congruent effects in all three cases (S6 Fig), a result supporting the conclusion that the two antibiotics induce giant cells by inhibition of their established targets.

### Survival assay

**Fig 7:**
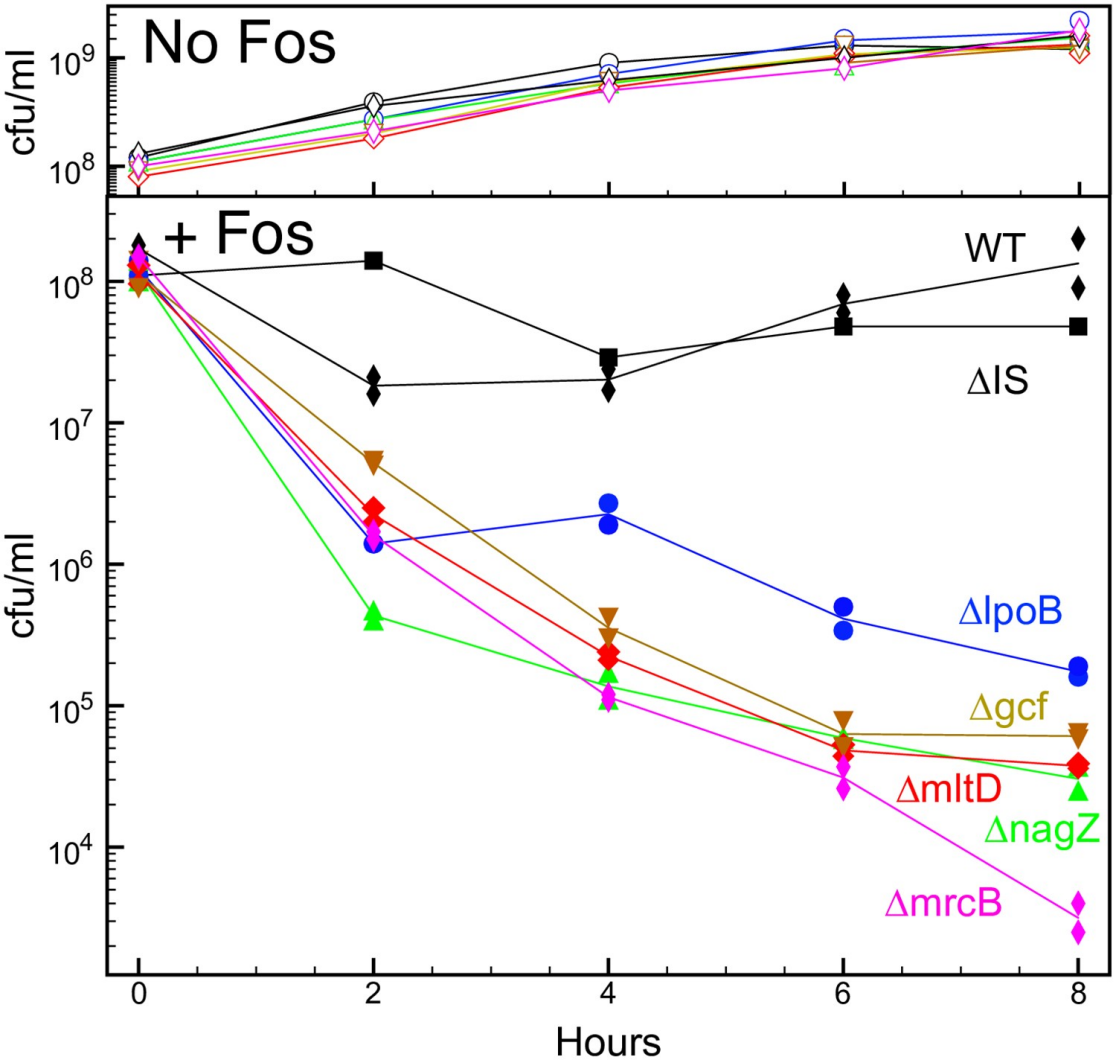
Fosfomycin killing of giant cell-minus mutants. The survival of bacteria in liquid protective medium following exposure to fosfomycin (192 μg/ml) was assayed. Wild type cells exposed to fosfomycin in liquid medium were smaller and less prone to lysis than on agar medium. The killing assays were carried out in duplicate for all strains except MAY116, and geometric means are plotted. The median geometric standard deviation for duplicates was 1.2, and in all cases was less than 1.8. WT, MAY101; ΔIS, MAY116; Δ*lpoB*, MAY114; Δ*gcf*, MAY108; Δ*mltD*, MAY110; Δ*nagZ*, MAY125; Δ*mrcB*, MAY113. cfu, colony-forming units; FOS, fosfomycin.

The mutations blocking fosfomycin-induced giant cell formation appeared microscopically to cause early, wholesale lysis (Fig 6). In order to quantify this lysis and death, we exposed bacteria grown in liquid protective medium to fosfomycin and followed recovery of viable bacteria (colony forming units) (Fig 7). Under these conditions, the wild type and a control mutant (carrying a neutral kanamycin resistance marker) exhibited good recovery for eight hours, whereas five mutants defective in forming giant cells all showed >100-fold reductions in recovery. The mutants and wild type showed comparable growth and survival in the absence of fosfomycin. The results indicate that unimpaired giant cell formation helps protect cells from rapid death when peptidoglycan precursor synthesis is blocked, i.e., contributes to fosfomycin tolerance.

### Giant cell formation in *Acinetobacter baumannii*

**Fig 8:**
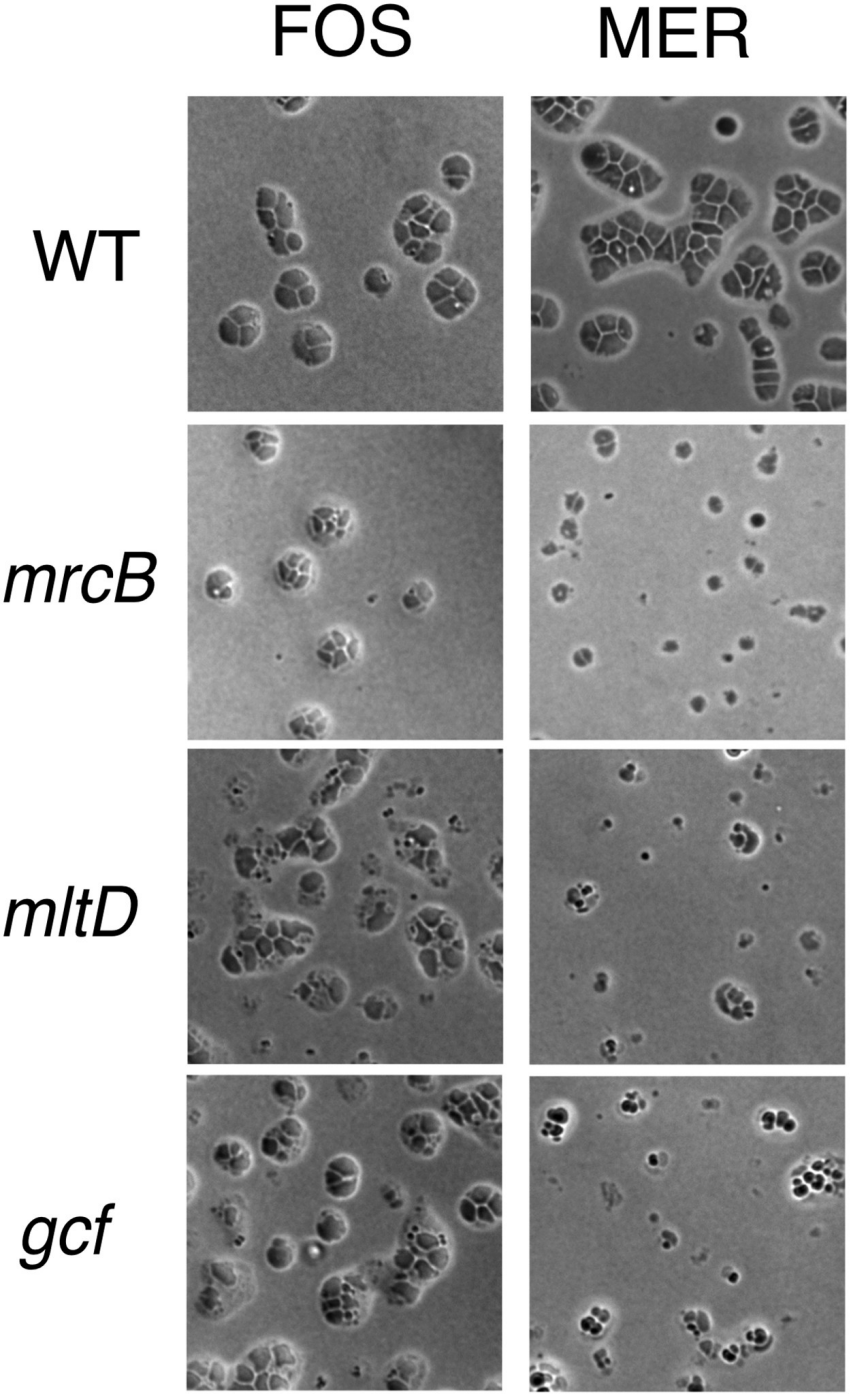
Giant cell formation by *A*. *baumannii*. *A*. *baumannii* strain AB5075 and the indicated transposon mutants were exposed to fosfomycin (192 μg/ml) (FOS) or meropenem (25 μg/ml) (MER) on protective agar and incubated for 8 hr at 37 °C. prior to imaging. WT, AB5075-UW; Δ*mrcB*, AB03662; *mltD*, AB07437; *gcf*, AB08926.

Is the response of *A*. *baylyi* to inhibition of peptidoglycan synthesis seen for other *Acinetobacter* species? A previous study by Doerr et al. found that meropenem treatment of a clinical isolate of the nosocomial pathogen *A*. *baumannii* converted the bacteria into non-dividing spheres that resemble small giant cells [16]. We examined giant cell formation by *A*. *baumannii* strain AB5075, and found that like *A*. *baylyi*, the strain formed giant cells upon exposure to fosfomycin or meropenem (Fig 8). In addition, transposon insertion mutants in *mrcB* (ABUW_1358), *mltD* (ABUW_2840) and *gcf* (ABUW_3408) interfered with the process in a manner similar to that seen for *A*. *baylyi*, albeit more weakly for fosfomycin induction (Fig 8). The finding that *A*. *baumannii* forms giant cells upon inhibition of peptidoglycan synthesis with some of the same genetic dependencies as *A*. *baylyi* suggests that the processes are similar in the two species.

### Giant cell formation pathway

**Fig 9:**
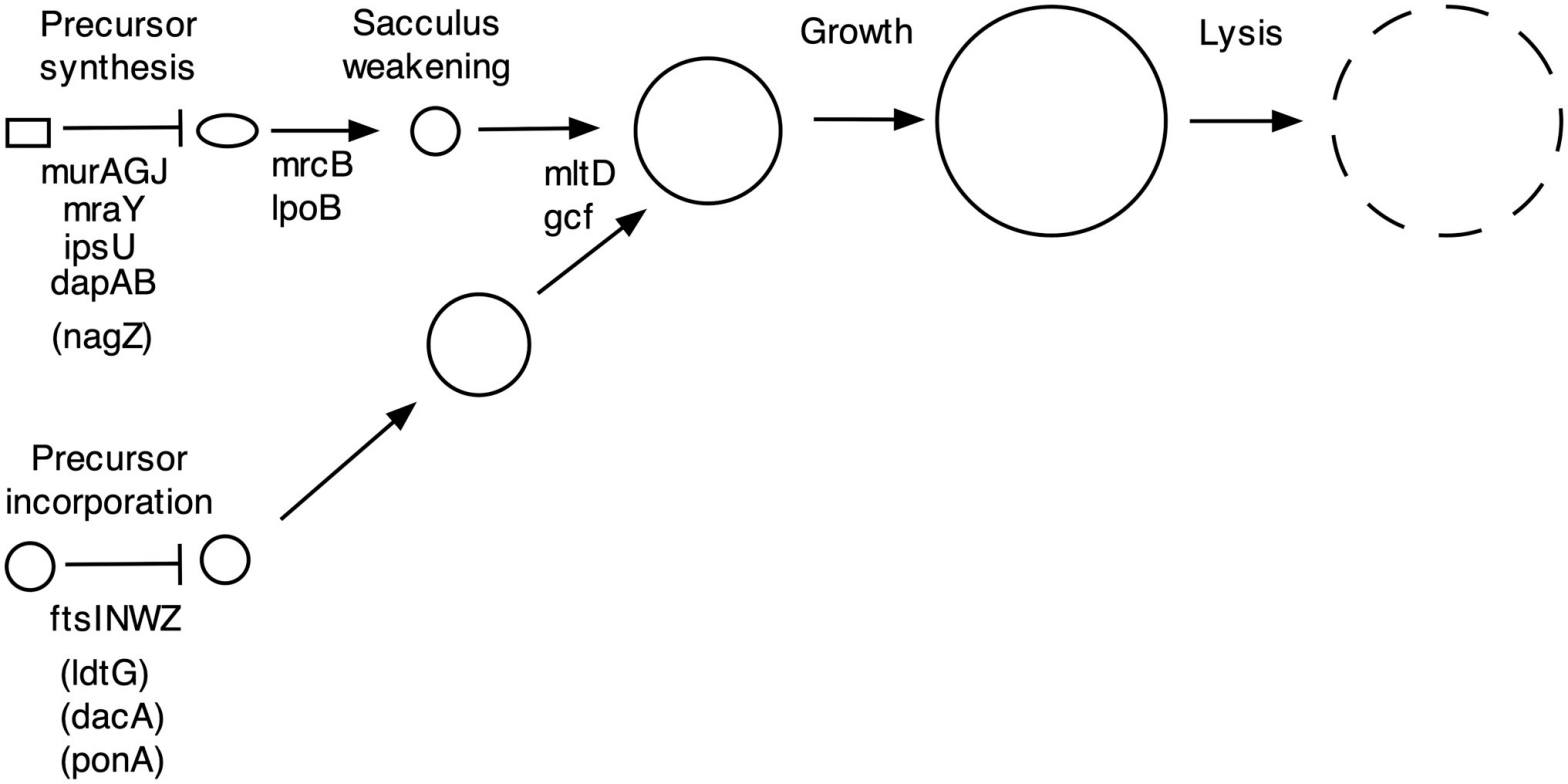
Giant cell formation pathway for *A*. *baylyi*. The pathway is initiated by mutations (or antibiotics) inactivating peptidoglycan precursor synthesis (upper branch) or polymerization (lower branch). The branches then converge with formation of giant cells and their eventual lysis. Mutations in genes in parentheses accelerate giant cell formation but do not induce it. Mutations that block the pathway at intermediate steps (*mrcB*, *lpoB*, *mltD* and *gcf*) also increase lysis.

Our results make it possible to formulate a genetic pathway for giant cell formation in *A*. *baylyi* (Fig 9). The pathway is initiated by blocking either the *de novo* biosynthesis of peptidoglycan precursor (upper left) or incorporation of the precursor into the existing murein sacculus (lower left), with convergence of the two initiating branches. The biosynthesis block can be achieved by null mutations in genes encoding biosynthetic enzymes or by an antibiotic that targets one of the enzymes (fosfomycin, inhibiting MurA). The precursor incorporation block can be achieved in the absence of elongation synthesis by null mutations in division genes or treatment with an antibiotic targeting septal peptidoglycan synthesis (aztreonam, inhibiting FtsI).

Other mutations accelerate giant cell formation but do not induce it. Inactivating a peptidoglycan recycling gene (*nagZ)* accelerates their formation and lysis after fosfomycin treatment, presumably because recycling provides an intermediate later in the precursor biosynthetic pathway than the product of the MurA step [36, 37]. Mutations inactivating three genes (*ldtG*, *dacA* and *ponA)* accelerate giant cell formation following inhibition of precursor incorporation (aztreonam treatment of an elongation-minus mutant). LdtG shows homology to enzymes (L,D-transpeptidases) that produce an unusual class of peptide cross-links (DAP-DAP) in peptidoglycan [38, 39], and reducing their level could accelerate giant cell formation by making cells more sensitive to loss of the more abundant cross-links (DAP-D-ala). DacA is a carboxypeptidase that is needed in *E*. *coli* to provide the substrate for L,D-transpeptidase [40], and may play an analogous role in *A*. *baylyi*. In *E*. *coli*, the transglycosylase activity of MrcB (PBP1B) is also required for L,D-transpeptidase-dependent growth, and the *ponA* (PBP1A) transglycosylase might play an analogous role in *A*. *baylyi*.

Mutations in four genes block giant cell formation following initiation. Two of the genes are relatively specific for the precursor synthesis branch (*mrcB* and *lpoB*), with mutants rounding up and lysing without enlarging significantly following initiation. The *mrcB* gene encodes a transglycosylase-transpeptidase (PBP1b), whereas the *lpoB* gene encodes an outer membrane protein that activates PBP1b [31]. In *E*. *coli*, PBP1b is needed for the generation of L forms [15, 41] and the conversion of spheroplasts to vegetative cells [42], indicating that the protein stabilizes peptidoglycan-deficient cells. The protein may thus also stabilize early intermediates in *A*. *baylyi* giant cell formation when precursor synthesis is blocked. Mutations in two other genes alter giant cell induction by both initiation branches of the pathway (*mltD*, *gcf*). However, the blocks are different for the two modes of initiation. When precursor synthesis is blocked, the mutants enlarge somewhat and then lyse. When precursor incorporation is blocked, they form small giant cells that also have a tendency to lyse. The *mltD* gene encodes a membrane lytic transglycosylase predicted to hydrolyze the peptidoglycan glycan backbone. MltD activity may contribute to giant cell formation by allowing the growing cytoplasm to emerge from the constraining murein sacculus when new peptidoglycan synthesis is blocked. We think it is unlikely that MltD carries out a function analogous to Slt in *E*. *coli* of selective elimination of uncross-linked precursors [11] because it is needed for giant cell induction when precursor synthesis is blocked. Gcf is a protein of unknown function, but is predicted to be an exported protein with a Sel1 tetratricopeptide protein interaction module [43]. Perhaps Gcf interacts with and activates MltD. A Δ*gcf* Δ*mltD* double mutant exhibits a giant cell induction defect no greater than that of a Δ*mltD* single mutant, supporting this possibility.

It remains to be determined how well the *Acinetobacter* pathway for giant cell formation represents the generation of wall-deficient forms of other bacteria. However, an intriguing potential link to the recovery of peptidoglycan-deficient *V*. *cholerae* spheroids is provided by the observation that a lytic transglycosylase (MltA) is required for the process [20].

### Giant cells *versus* L forms

What is the relationship between giant cells and L forms? Both types of cells result from inhibition of peptidoglycan synthesis and are pleomorphic, but L forms proliferate and giant cells do not. We hypothesize that giant cells represent a primary consequence of growth without peptidoglycan synthesis, and that additional mutations are required for them to proliferate as L forms. This model readily accounts for overlap in functions needed for production of the two types of cells (e.g., *mrcB*) and the low yield of L forms when peptidoglycan synthesis is inhibited (e.g., ~10^−5^–10^−4^/cell) [41]. Natural variation in the capacity of different bacteria to generate L forms may reflect differences in other factors needed for L form growth, e.g., the nature and amount of polysaccharide capsule [41, 44].

### Conclusion

In this study we used a new procedure for examining the terminal phenotypes of bacteria deleted of essential genes to analyze mutations disrupting peptidoglycan synthesis. Mutations blocking the process in different ways led to the formation of pleomorphic giant cells, and the phenotypes of mutants defective in making the unusual cells suggested a genetic pathway for their formation.

## Materials and methods

### Strains and growth media

Mutant strains were derivatives of *Acinetobacter baylyi* ADP1 (MAY101) [3] (the gift of C. Harwood) and *A*. *baumannii* AB5075-UW [45]. *A*. *baylyi* MAY106 (“ΔE”) (*ΔpbpA ΔrodA ΔponA*) is an unmarked peptidoglycan elongation-deficient triple mutant constructed from ADP1. *A*. *baylyi* MAY116 is a “wild type” control strain carrying a kanamycin resistance marker (*nptII*) in place of an IS element (IS1236_1) [3, 24]. MAY103 carries a Δ*pbpA* allele marked with *nptII*. A complete list of strains is provided in S2 Database.

Growth media were TYE (10 g tryptone, 5 g yeast extract, 8 g sodium chloride and 15 g agar per liter), LB (TYE lacking agar) and minimal-succinate (M9 medium [46] supplemented with 15 mM sodium succinate, 2 mM magnesium sulfate, 0.1 mM calcium chloride and 1–3 μM ferrous sulfate ±15 g agar/l. Protective medium was minimal-succinate supplemented with 0.4 M sucrose and 10 mM magnesium sulfate, and was solidified with 1.5% agar (“protective agar”) or 2% agarose (“protective agarose”). Supplements were routinely used at the following concentrations: kanamycin, 10 μg/mL (TYE) or 20μg/mL (minimal media); fosfomycin, 192–360 μg/mL; aztreonam, 120–190 μg/mL; and meropenem, 5–25 μg/mL. *A*. *baylyi* strains were routinely grown at 30° C whereas *A*. *baumannii* strains were grown at 37° C.

### Construction of deletion mutations

We created deletion mutations by natural transformation of linear DNA fragments constructed by PCR using extension overlap [22, 47, 48]. The transformed fragments carried sequences of homology of ~2 kb flanking targeted genes that were either directly joined (for unmarked deletions) or flanked a kanamycin resistance determinant (for *kan*-marked deletions). Unmarked deletions were in-frame and included 18 bp insertions carrying diagnostic restriction sites at the deletion junctions. Marked deletions carried the *nptII* gene (“*kan*”) from plasmid pACYC177 [49] encoding kanamycin resistance, with *nptII* in the same orientation as the deleted gene. In creating deletions, we designed primers (and often employed the same primers) as described previously [25] (S2 Database). Thermocycling reactions employed Q5 Polymerase (New England Biolabs), and DNA fragments were routinely purified using Qiaquick columns (Qiagen) prior to transformation.

The mutagenic DNA fragments were transformed into *A*. *baylyi* cultures grown overnight in minimal-succinate with 1uM ferrous sulfate, diluted 1:5 in fresh medium and grown one hour with shaking at 30°. DNA was added at 1μg/mL, followed by incubation for 3 hours with shaking and plating on selective (marked deletions) or non-selective (unmarked deletions) media. Unmarked deletion mutations were identified by screening individual colonies by PCR using primers flanking targeted genes. Essential gene *kan*-marked deletion mutations were selected by plating on protective medium containing kanamycin (20 μg/mL). All unmarked and the majority of marked deletion mutations were verified by PCR. Microcolonies of the deletion mutants were generally imaged after 18–24 hours incubation at 30° C. In a typical experiment creating *kan*-marked essential gene deletions, 5–10% of the cells were transformed forming microcolonies of cells carrying the deletion. There was commonly a background of ~10^−6^ fast-growing colonies that carried both deleted and undeleted versions of the targeted genes [25], presumably arising from cells with tandem duplications.

### Microscopy

Bacterial microcolonies were routinely imaged after growth on protective agar in 15 X 60 mm diameter Petri plates under bright field illumination using a Nikon Eclipse 90i with an ELWD 20X objective equipped with 2X digital zoom. For high-resolution imaging, microcolonies were grown on thin 2% agarose protective medium pads under cover slips in Gene Frames (Thermo Scientific) prior to phase contrast imaging using a 100X oil immersion objective. The microcolonies of giant cells grown in Gene Frames tended to be somewhat smaller and lyse somewhat earlier than those formed on plates.

### Transposon mutagenesis

ADP1 was mutagenized by insertion of the tetracycline resistance-marked transposon T26 using a modification of a previously described procedure [45]. An overnight LB culture of ADP1 was diluted 1:200 into fresh medium without NaCl and grown with shaking to OD600 0.8. Cells were then pelleted and washed three times in decreasing volumes of cold 10% glycerol until cells had been concentrated approximately 150-fold. Aliquots (~0.5 μL) of transposon-transposase complex were mixed with 50 μl concentrated cells for electroporation (1.8kV, 200 Ω, 25μF using a Biorad Gene Pulser). Insertion mutants were selected on TYE media supplemented with tetracycline (5–7.5 μg/mL) by overnight growth at 30°, and then harvested and pooled. Two independent pools were created, each made up of approximately 80,000 independent mutants. A *pbpA*-minus transposon mutant pool was created by transforming one of the ADP1 mutant pools with a PCR fragment corresponding to the Δ*pbpA*::*kan* mutation found in MAY103, with selection for kanamycin resistance on minimal-succinate agar.

### Tn-seq analysis

Tn-seq screens were carried out for cells grown on fosfomycin or aztreonam. For the fosfomycin screen, one of the ADP1 transposon mutant pools was plated on protective medium supplemented with 360 μg/mL fosfomycin at approximately 5x10^7^ and 5x10^8^ cells/plate, grown for 24 hours at 30°C and harvested (two Tn-seq assays total). For the aztreonam Tn-seq screen, the Δ*pbpA*::*kan* transposon mutant pool was plated on protective medium supplemented with aztreonam (120 or 192 μg/mL) at ~5X10^5^ and 1X10^7^ cells/plate, and cells were harvested at 24 and 48 hours (eight Tn-seq assays total). As controls, mutant pools were grown on protective medium lacking antibiotic. Tn-seq analysis was carried out using a terminal deoxynucleotidyl terminal transferase-based procedure [45].

To identify genes whose inactivation affected giant cell formation after fosfomycin or aztreonam treatment, combined read counts for insertions in nonessential genes (5 to 90% of predicted coding regions, normalized for total reads/sequencing run) were evaluated for each time point analyzed, and histograms of the ratios of the log2-transformed read counts of antibiotic-treated to the corresponding antibiotic-untreated cultures plotted. Genes whose mutants were significantly depleted or enriched under giant cell induction conditions were identified using normal distribution thresholds specified for each condition in Data Set S1, with genes identified in multiple independent experiments chosen for subsequent validation studies using constructed deletion mutations.

## Supporting information

S1 Fig Giant cell morphology. **A.**, paired phase contrast and fluorescence images of giant cells of a strain expressing cytoplasmic green fluorescent protein (MAY118 or MAY119). **B.**, giant cells with peripheral wispy filaments and vesicles. Giant cells were induced by exposure of wild type (MAY101) to fosfomycin (192 μg/ml) for 20–24 h on protective agar and then suspended in protective medium for microscopy. Scale bar, 10 μm. Fluorescent imaging employed an EGFP/FITC/CY2/Alexa Fluor 488 Filter Set.
(TIFF)

S2 Fig Mutations accelerating giant cell formation after aztreonam treatment. Deletions of three genes (Δ*dacA* (MAY111), Δ*ldtG* (MAY115) and Δ*ponA* (MAY105)) in a ΔPBP2 genetic background speed giant cell formation and lead to premature lysis on protective agar. Note that the three mutants have larger cells that the ΔPBP2 parent strain (MAY102) at 9 h, and have mostly lysed by 24 h. A Δ*mrcB* mutant (MAY113) exhibits relatively normal giant cell formation under these conditions. The *dacA*, *ldtG* and *ponA* deletions reduced the aztreonam minimal inhibitory growth concentration 3–8 fold. Scale bar, 10 μm.
(TIFF)

S3 Fig Blocking peptidoglycan recycling accelerates giant cell formation and lysis. The formation of giant cells induced by exposure to different antibiotics in protective agar is shown for a wild type control strain (ΔIS) (MAY116) and a mutant deleted of *nagZ*, a gene required for peptidoglycan recycling (encoding ß-N-acetyl-glucosaminidase) (MAY125). The mutation accelerates formation and lysis of giant cells upon fosfomycin treatment, and causes smaller but detectable increases in lysis at 24 h in the aztreonam and meropenem treatment conditions. The Δ*nagZ* mutation reduced the fosfomycin MIC four-fold. Scale bar, 10 μm.
(TIFF)

S4 Fig ZipA^-^ PBP2^-^ double mutant cells are viable giant cell producers. Microcolonies of *zipA*^+^ and **Δ***zipA* cells with or without the PBP2 gene (*pbpA*) are shown after growth for 24 h on protective agarose pads. WT, MAY101; Δ*pbpA*, MAY102; Δ*zipA*::*kan*, MAY130; Δ*pbpA* Δ*zipA*::*kan*, MAY131. Scale bar, 10 μm.
(TIFF)

S5 Fig Giant cell formation by LOS-minus mutants. Microcolones of bacteria grown 12 hours on protective agarose pads with and without fosfomycin (192 μg/ml) are shown. The wild-type control strain (MAY116) carries IS1236 1::*kan*. Scale bar, 10 μm.
(TIFF)

S6 Fig Reverse phenocopy test. The figure compares the microcolonies of giant cells formed in response to fosfomycin and aztreonam treatment compared to deletion of their presumptive target genes (*murA* and *ftsI* respectively) in the absence of the antibiotics. Bacteria (MAY107, MAY109 and MAY112) were grown 24 hr, 30 °C on protective agar in the presence of fosfomycin (360 μg/ml) or aztreonam (192 μg/ml), or for 18 h, 30 °C following transformation with selection on protective agar with 20 μg/ml kanamycin to create the indicated deletion mutants. Scale bar, 10 μm.
(TIFF)

S1 Movie Giant cell formation after deletion of *murA*. The field shows the growth two cells for 10 hours after spotting a wild type (MAY101) transformation mix with selection for a Δ*murA*::*kan* on protective agarose pads. The cell at the center appears to have obtained the mutagenic PCR fragment and is thus kanamycin resistant, whereas that at the upper right has not and growth is inhibited by the kanamycin. The center cell divides and gives off cells that enlarge into amorphous giant cells. The microcolony that forms also contains cells that retain their normal size and shape and are presumably kanamycin sensitive. These cells may originate from transformants with multiple chromosomes that segregate both mutant and wild type chromosomes. Some of the giant cells lyse, while others grow in an amorphous amoeboid fashion. Many of the giant cells show small membranous filaments and small vesicles at their surfaces. Imaging was conducted using a Nikon Ti-E inverted wide-field fluorescence microscope with a large format sCMOS camera (Andor NEO) and controlled by NIS-Elements. Following transformation, cells were inoculated onto 2% agarose pads made with protective minimal-succinate medium containing kanamycin (20 μg/ml) to select growth of cells carrying the deletion insert. Cells were imaged using brightfield illumination at 30° every 2 min for 10 hours, and images used to generate time-lapse videos of micro-colony development.
(MP4)

S1 Table Deletion mutant giant cell formation. (DOCX)

S1 Database Genes depleted in fosfomycin Tn-seq of wild-type. (XLSX)

S2 Database Bacterial strains and primers. (XLSX)
