# Supplementary figures and images for: Essential gene deletions producing gigantic bacteria
Source: PLoS Genet. 2019 Jun 10;15(6):e1008195. doi: 10.1371/journal.pgen.1008195 (PMC6586353; doi:10.1371/journal.pgen.1008195)

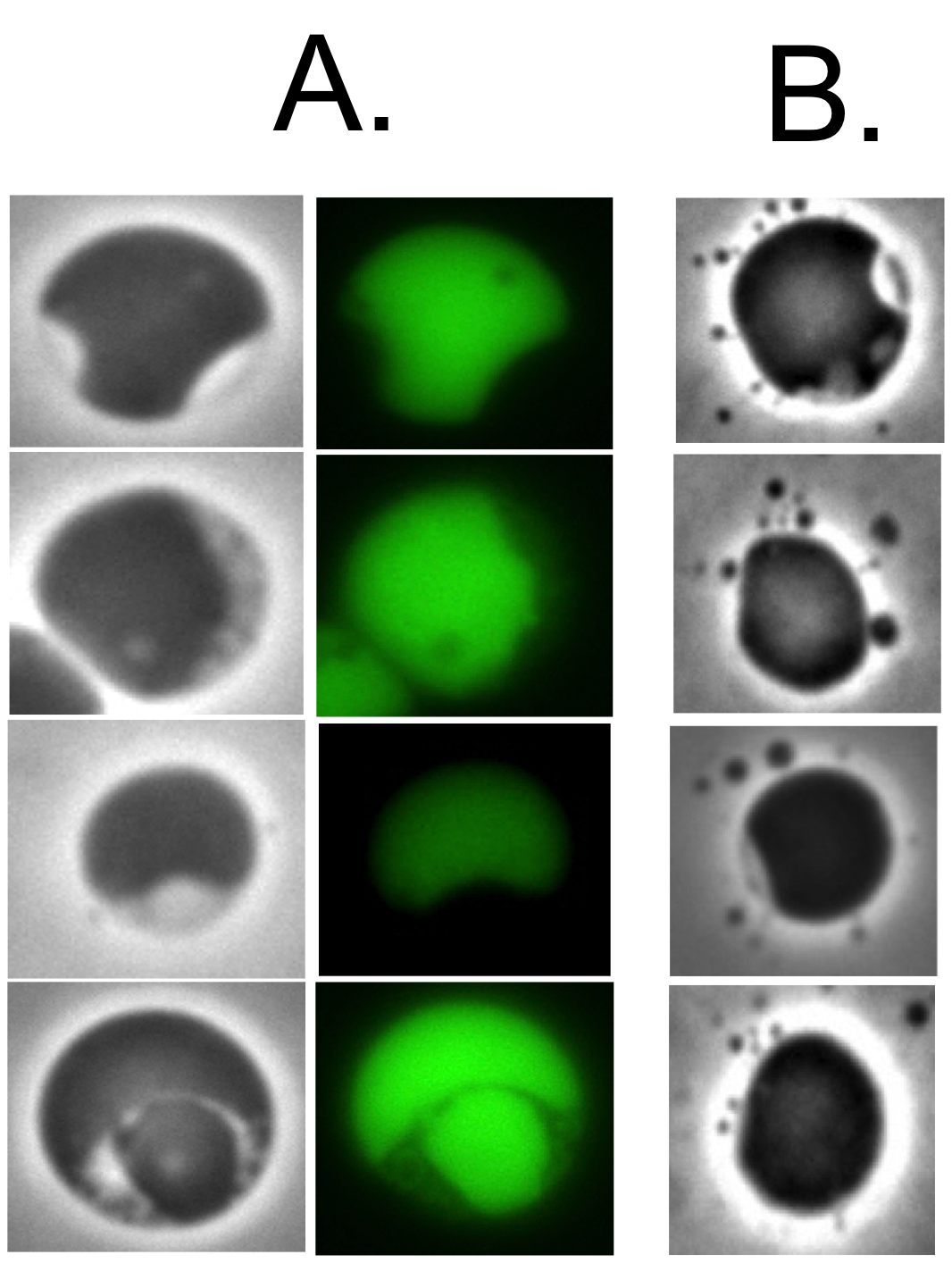

Supplement: S1 Fig — A., paired phase contrast and fluorescence images of giant cells of a strain expressing cytoplasmic green fluorescent protein (MAY118 or MAY119). B., giant cells with peripheral wispy filaments and vesicles. Giant cells were induced by exposure of wild type (MAY101) to fosfomycin (192 μg/ml) for 20–24 h on protective agar and then suspended in protective medium for microscopy. Scale bar, 10 μm. Fluorescent imaging employed an EGFP/FITC/CY2/Alexa Fluor 488 Filter Set. (TIFF) [file pgen.1008195.s001.tiff]

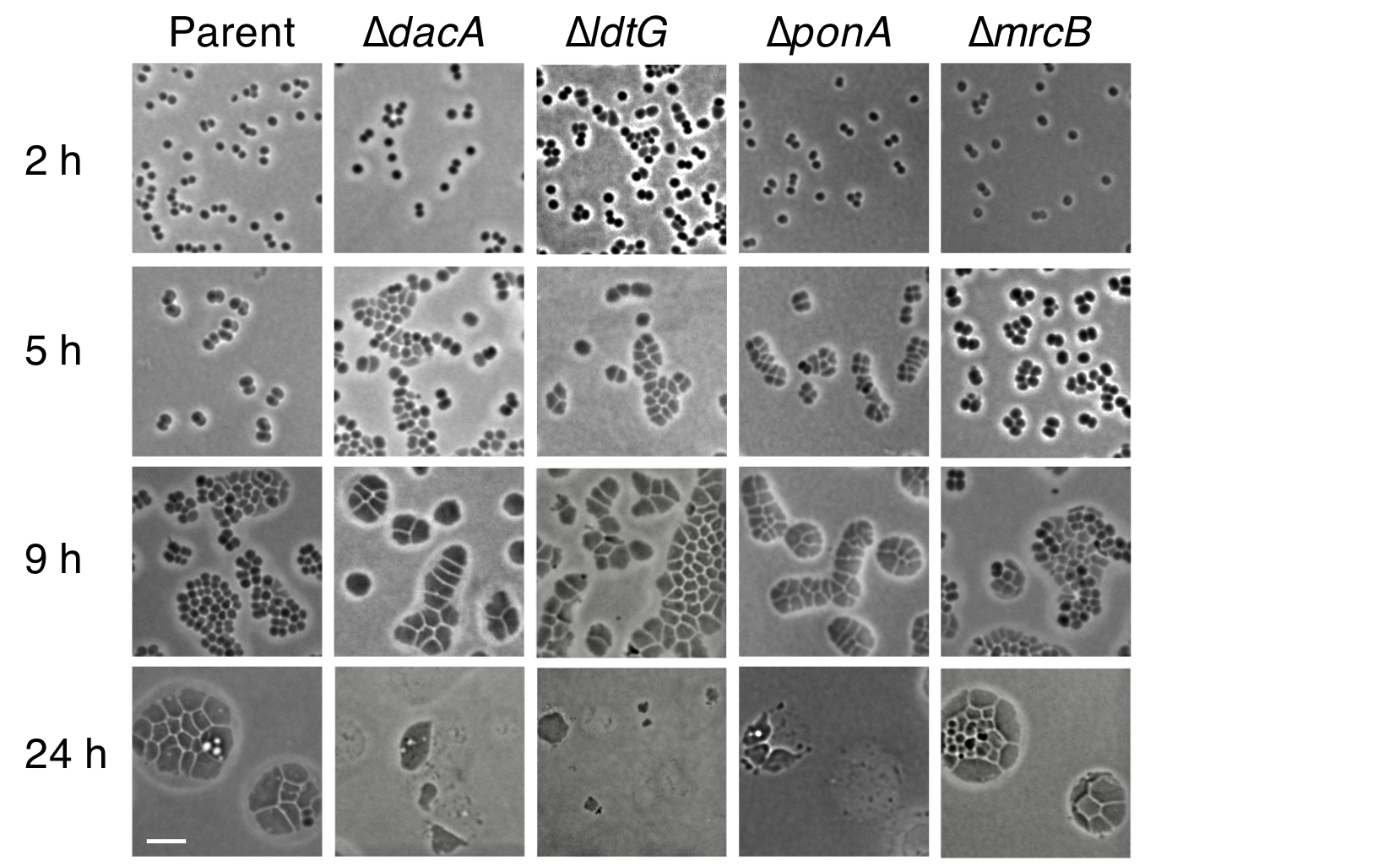

Supplement: S2 Fig — Deletions of three genes (ΔdacA (MAY111), ΔldtG (MAY115) and ΔponA (MAY105)) in a ΔPBP2 genetic background speed giant cell formation and lead to premature lysis on protective agar. Note that the three mutants have larger cells that the ΔPBP2 parent strain (MAY102) at 9 h, and have mostly lysed by 24 h. A ΔmrcB mutant (MAY113) exhibits relatively normal giant cell formation under these conditions. The dacA, ldtG and ponA deletions reduced the aztreonam minimal inhibitory growth concentration 3–8 fold. Scale bar, 10 μm. (TIFF) [file pgen.1008195.s002.tiff]

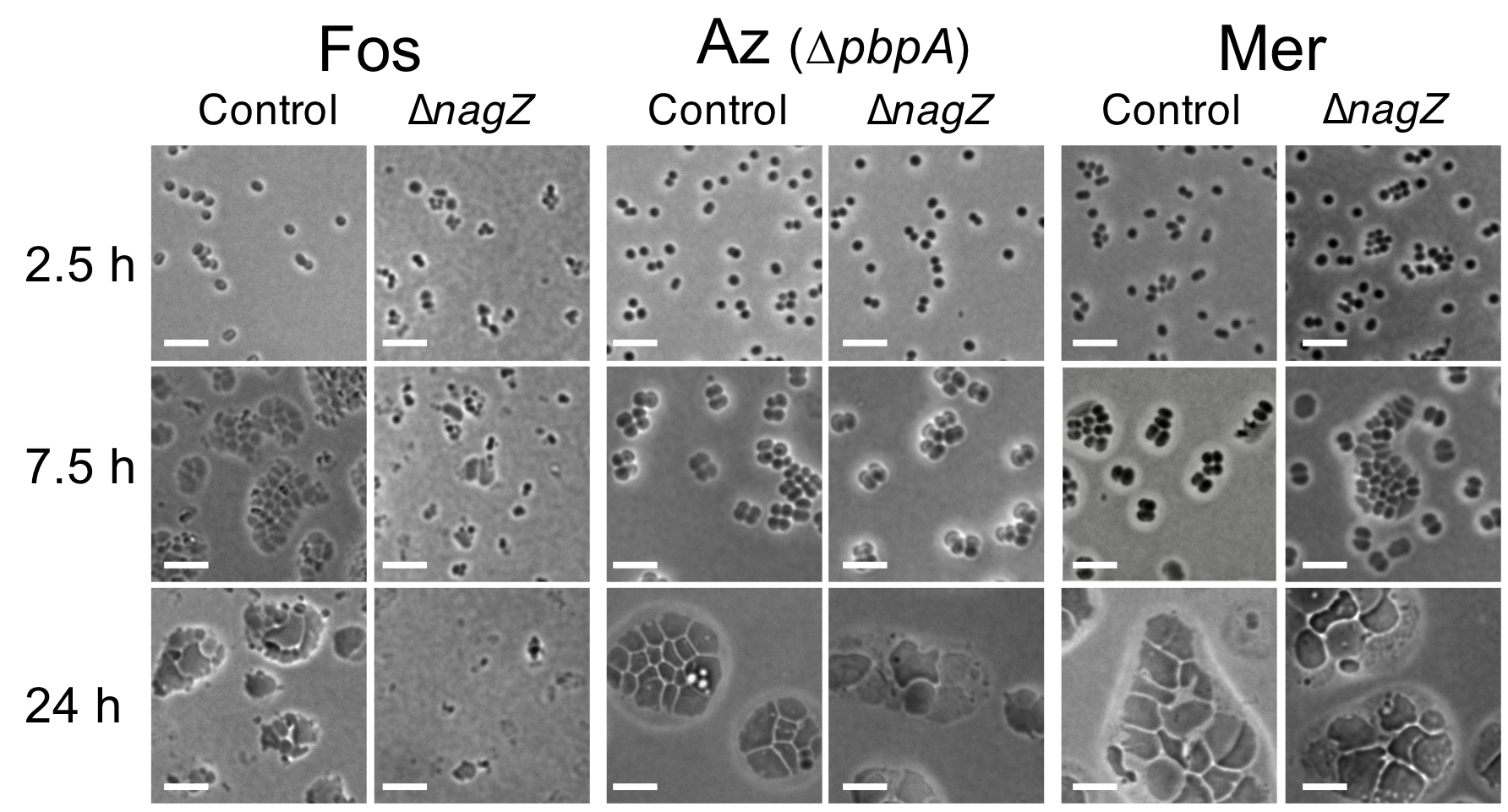

Supplement: S3 Fig — The formation of giant cells induced by exposure to different antibiotics in protective agar is shown for a wild type control strain (ΔIS) (MAY116) and a mutant deleted of nagZ, a gene required for peptidoglycan recycling (encoding ß-N-acetyl-glucosaminidase) (MAY125). The mutation accelerates formation and lysis of giant cells upon fosfomycin treatment, and causes smaller but detectable increases in lysis at 24 h in the aztreonam and meropenem treatment conditions. The ΔnagZ mutation reduced the fosfomycin MIC four-fold. Scale bar, 10 μm. (TIFF) [file pgen.1008195.s003.tiff]

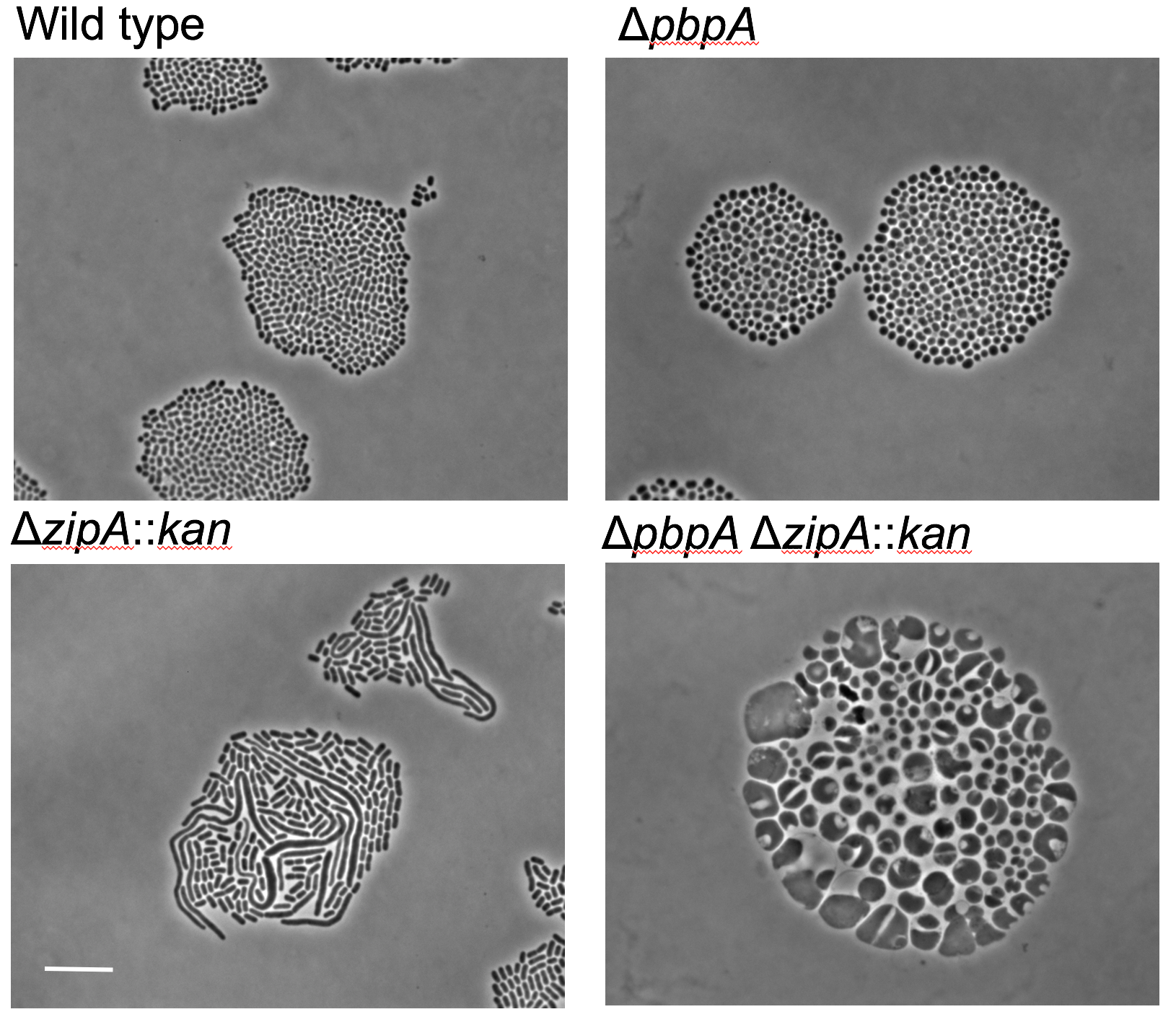

Supplement: S4 Fig — Microcolonies of zipA+ and ΔzipA cells with or without the PBP2 gene (pbpA) are shown after growth for 24 h on protective agarose pads. WT, MAY101; ΔpbpA, MAY102; ΔzipA::kan, MAY130; ΔpbpA ΔzipA::kan, MAY131. Scale bar, 10 μm. (TIFF) [file pgen.1008195.s004.tiff]

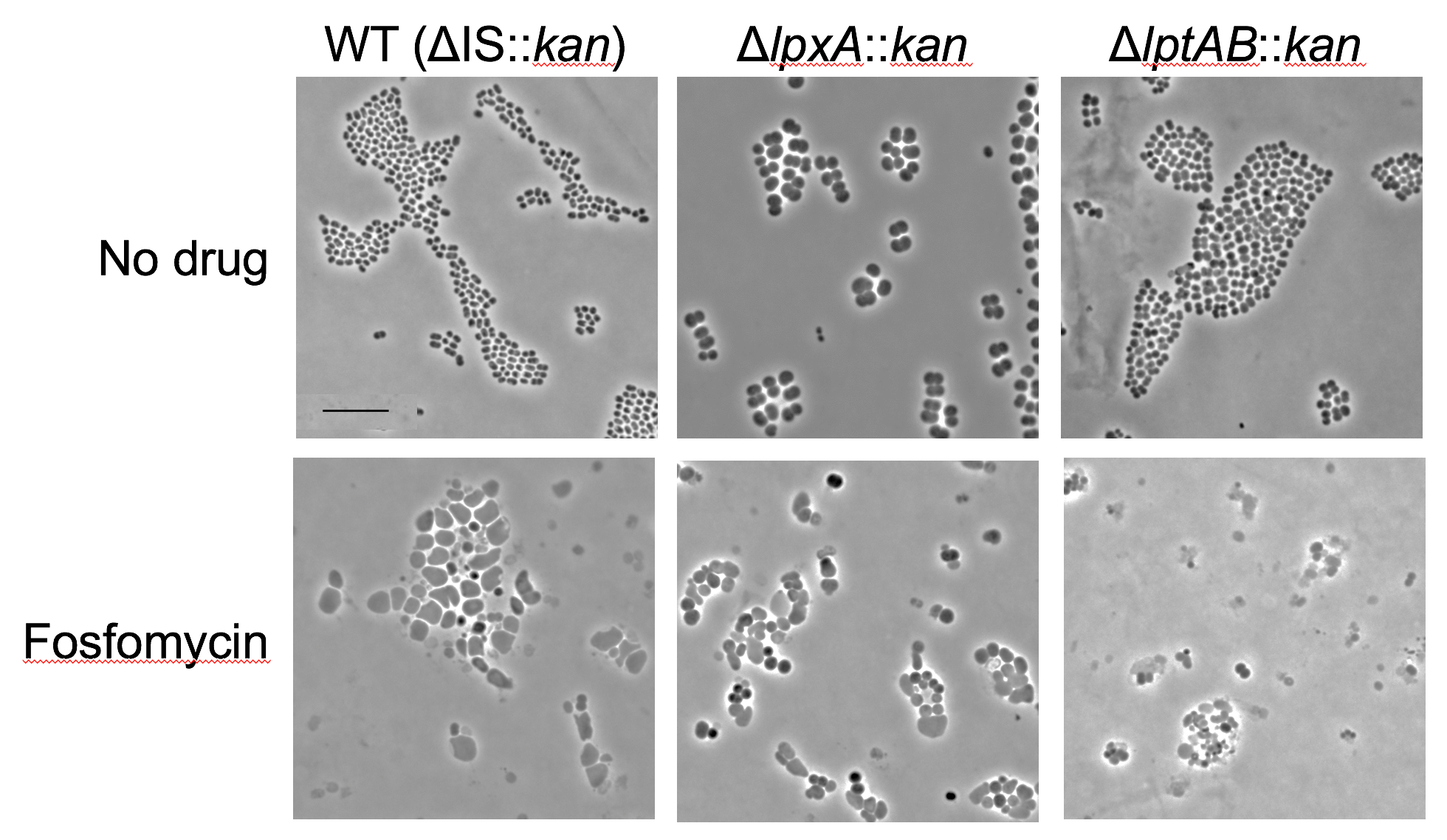

Supplement: S5 Fig — Microcolones of bacteria grown 12 hours on protective agarose pads with and without fosfomycin (192 μg/ml) are shown. The wild-type control strain (MAY116) carries IS1236 1::kan. Scale bar, 10 μm. (TIFF) [file pgen.1008195.s005.tiff]

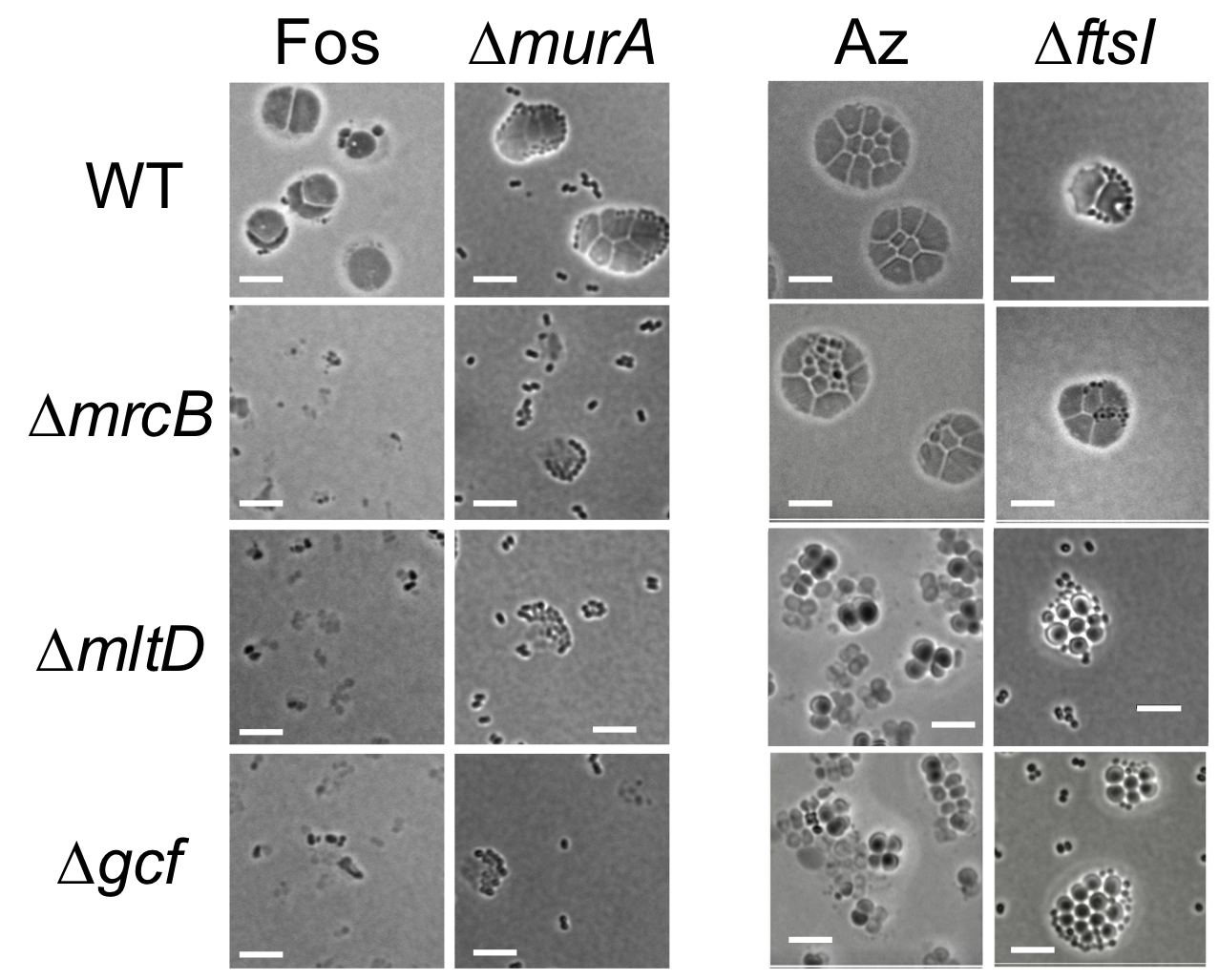

Supplement: S6 Fig — The figure compares the microcolonies of giant cells formed in response to fosfomycin and aztreonam treatment compared to deletion of their presumptive target genes (murA and ftsI respectively) in the absence of the antibiotics. Bacteria (MAY107, MAY109 and MAY112) were grown 24 hr, 30 °C on protective agar in the presence of fosfomycin (360 μg/ml) or aztreonam (192 μg/ml), or for 18 h, 30 °C following transformation with selection on protective agar with 20 μg/ml kanamycin to create the indicated deletion mutants. Scale bar, 10 μm. (TIFF) [file pgen.1008195.s006.tiff]
