# Supplementary material for: Essential gene deletions producing gigantic bacteria
Source: PLoS Genet. 2019 Jun 10;15(6):e1008195. doi: 10.1371/journal.pgen.1008195 (PMC6586353; doi:10.1371/journal.pgen.1008195)
Supplement: S1 Table — (DOCX) [file pgen.1008195.s008.docx]

| **Table S1. Deletion mutant giant cell formation.** The phenotypes seen under giant cell induction conditions for deletion mutants of candidate genes are provided. Strong phenotypes and the corresponding genes are indicated in boldface type. Growth on protective medium without antibiotic was assessed by visual inspection. PBP, penicillin binding protein; nd, not determined; n/a, not applicable | | | | | | | | | |  |
| --- | --- | --- | --- | --- | --- | --- | --- | --- | --- | --- |
|  |  |  |  |  | **Tn-seq Recovery** | | **Single mutant giant cell phenotype** | | |  |
| **Locus** | **Strain (MAY #)** | **Gene** | **Product** | **Growth** | **Fosfomycin** | **Aztreonam** | **Fosfomycin**  **(wt)** | **Aztreonam (∆PBP2)** | **Meropenem**  **(wt)** | |
| ACIAD0020 | 120 | fkpB | Peptidyl-prolyl cis-trans isomerase | +++ | Increased  (2/2) | nd | Late lysing | nd | nd | |
| ACIAD0196 | 121 | ycfQ | Repressor of bshA (ycfR) | +++ | Increased  (2/2) | Increased  (6/8) | Forms giant cells | Antibiotic resistant | nd | |
| **ACIAD0251** | 122 | **blhA** | β-lactam hypersensitive | +++ | Decreased (1/2) | Decreased (5/8) | Early forming, early lysing | Forms giant cells | **Early forming, early lysing** | |
| ACIAD0262 | 123 | pbpG | D-alanyl-D-alanine endopeptidase | ++ | Increased  (2/2) | nd | Forms giant cells | Forms giant cells | nd | |
| ACIAD0273 | 124 | dksA | Stringent response regulator | +/- | Increased (2/2) | Essential | Mostly defective | Inefficient, blocked as intermediates | Defective | |
| **ACIAD0527** | 107, 108 | **gcf** | Sel1 domain | +++ | Decreased (2/2) | nd | **Fails, early lysing** | **Blocked as intermediate, early lysing** | **Blocked as intermediate, early lysing** | |
| **ACIAD0551** | 125 | **nagZ** | N-acetyl-beta-glucosaminidase | +++ | Decreased (2/2) | Decreased (7/8) | **Early forming,**  **Early lysing** | Early lysing | Forms giant cells | |
| ACIAD0552 | 126 | prc | Periplasmic protease | ++ | – | Decreased (8/8) | Late forming | Early forming, late lysing | Late forming, early lysing | |
| ACIAD0657 | 127 | rpoN | RNA polymerase sigma-54 factor | ++ | Increased  (2/2) | – | Late forming | nd | nd | |
| ACIAD0703 | 128 | typA | GTP-binding elongation factor family | + | Increased  (2/2) | Essential | Late forming,  early lysing | nd | nd | |
| ACIAD0795 | 129 | - | L, D-transpeptidase family | +++ | – | – | Early lysing | nd | Antibiotic resistant | |
| ACIAD0847 | 130 | zipA | Cell division protein | +++ | Decreased (2/2) | nd | Early lysing | Early forming, early lysing | Forms giant cells | |
| ACIAD1082 | 132 | - | Transcriptional regulator | - | Increased  (2/2) | Essential | Late forming | More resistant | nd | |
| ACIAD1101 | 102, 103 | pbpA | PBP2 | ++ | – | n/a | Forms giant cells | Forms giant cells | Forms giant cells | |
| ACIAD1123 | 133 | zapA | Cell division | +++ | – | nd | Forms giant cells | Forms giant cells | Forms giant cells | |
| **ACIAD1138** | 109, 110 | **mltD** | Lytic transglycosylase | ++ | Decreased (2/2) | – | **Fails, early lysing** | **Blocked as intermediate, early lysing** | **Blocked as intermediate, early lysing** | |
| **ACIAD1225** | 111 | **dacA** | D-ala-D-ala carboxypeptidase (PBP5) | ++ | – | Decreased (8/8) | Forms giant cells | **Early forming, early lysing** | **Early lysing** | |
| ACIAD1229 | 134 | ΔnlpD |  | +++ | - | Decreased (4/8) | Forms giant cells | Forms giant cells | Forms giant cells | |
| **ACIAD1396** | 135 | - | Histidine triad | ++ | Increased  (2/2) | Increased  (8/8) | **Late lysing** | Antibiotic resistant | nd | |
| ACIAD2029 | 136 | prmB | Protein- methylase | - | Increased  (2/2) | Increased  (8/8) | Defective | nd | Late forming | |
| **ACIAD2234** | 112, 113 | **mrcB** | PBP1b | +++ | Decreased (2/2) | Decreased (2/8) | **Fails, early lysing** | Early forming, early lysing | **Defective,**  **early lysing** | |
| **ACIAD2235** | 114 | **lpoB** | PBP1b activator | +++ | Decreased (2/2) | Decreased (1/8) | **Fails, early lysing** | Forms giant cells | Forms giant cells | |
| ACIAD2263 | 104 | rodA | SEDS family | ++ | – | Decreased (1/8) | Forms giant cells | nd | Forms giant cells | |
| ACIAD2264 | 137 | mltB | Lytic transglycosylase | +++ | – | Decreased (2/8) | Forms giant cells | Forms giant cells | Forms giant cells | |
| **ACIAD2336** | 138 | **zapE** | Divisome ATPase | + | Increased  (2/2) | Increased  (8/8) | **Late forming, late lysing** | Antibiotic resistant | Late lysing | |
| **ACIAD2475** | 115 | **ldtG** | L, D-Transpeptidase | +++ | – | Decreased (8/8) | Forms giant cells | **Early forming, early lysing** | **Early lysing** | |
| ACIAD2561* | 139 | obg | GTP-binding | ++ | Increased (2/2) | Increased (4/8) | Forms giant cells | nd | Forms giant cells | |
| **ACIAD2589** | 140 | **mltG** | Peptidoglycan terminase | +++ | - | Decreased  (2/8) | Forms giant cells | nd | **Early lysing** | |
| ACIAD2661 | 141 | lppL | Lipoprotein | ++ | Increased (2/2) | Increased  (4/8) | Early lysing | Forms giant cells | Forms giant cells | |
| ACIAD2832 | 142 | - | Lytic transglycosylase | +++ | – | nd | Forms giant cells | Forms giant cells | Early lysing | |
| ACIAD2894 | 143 | smpB | SsrA-binding protein | ++ | Increased  (2/2) | Increased  (8/8) | Forms giant cells | nd | nd | |
| ACIAD3119 | 144 | engC | GTPase | ++ | Increased  (2/2) | Increased  (8/8) | Late forming, late lysing | nd | nd | |
| **ACIAD3361** | 104, 105 | **ponA** | PBP1a | +++ | – | Decreased (7/8) | Forms giant cells | **Early forming, early lysing** | Forms giant cells | |
| **ACIAD3380** | 145 | **ettA** | Translation regulator | + | Increased  (2/2) | Increased  (5/8) | **Late forming, late lysing** | Antibiotic resistant | Antibiotic resistant | |
| ACIAD3508 | 146 | mepM | Murein DD-endopeptidase | +++ | – | Decreased (1/8) | Forms giant cells | Forms giant cells | Early lysing | |
| ACIAD3549 | 147 | gshA | Glutamate--cysteine ligase | +/- | Increased  (2/2) | Increased  (8/8) | Very late forming | nd | nd | |
| ACIAD3068, ACIAD3326 | 148 | relA spoT | ppGpp synthetase,  hydrolase | +++ | -- | -- | Small giant cells (irregularly shaped) | Early forming Small giant cells  (irregularly shaped) | Small giant cells (irregularly shaped) | |
| ACIAD1101, 2263, 3361 | 106 | pbpA, rodA, ponA | Peptidoglycan elongation functions | ++ | n/a | n/a | Forms giant cells | Early forming, early lysing | Early lysing | |

*Kanamycin resistance-marked, partial deletion of 204 bp from the 3’end of the gene. The resulting protein should be similar to the truncated protein presumably made by the transposon mutant that accounted for enrichment of this essential gene in the Tn-seq. See Database S2 for more information.
